# Genetically Proxied Antidiabetic Drug Target and Primary Open‐Angle Glaucoma: A Mendelian Randomization Study

**DOI:** 10.1002/hsr2.70162

**Published:** 2024-10-24

**Authors:** Kefu Tang, Wenqiu Wang, Weiteng Chang, Xi Wu

**Affiliations:** ^1^ Department of Clinical Laboratory, Prenatal Diagnosis Center, Changning Maternity and Infant Health Hospital East China Normal University Shanghai China; ^2^ Department of Ophthalmology, Shanghai General Hospital Shanghai Jiao Tong University School of Medicine Shanghai China; ^3^ Department of Ophthalmology, NHC Key Laboratory of Myopia, Shanghai Research Center of Ophthalmology and Optometry, Eye & ENT Hospital Fudan University Shanghai China; ^4^ Key Laboratory for the Genetics of Developmental and Neuropsychiatric Disorders (Ministry of Education), Bio‐X Institutes Shanghai Jiao Tong University Shanghai China

**Keywords:** ABCC8, antidiabetics drug, Mendelian randomization, primary open‐angle glaucoma

## Abstract

**Background and Aims:**

Observational studies suggest that antidiabetic drugs may lower POAG risk; while the causal relationship remains unclear. Naturally occurring variation in genes encoding antidiabetics drug targets can be used as proxies to investigate long‐term therapeutic effect of these drugs on POAG risk.

**Methods:**

We performed a two‐sample Mendelian randomization study to evaluate the potential effect of antidiabetic drug targets on POAG in Europeans and East Asians. To proxy antidiabetic drugs (ABCC8, PPARG, GLP1R, SLC5A2), we leveraged genetic variants located near or within drug target genes that were associated with HbA1c. The validity of our ancestry‐specific genetic instrument was checked with multipul positive control outcomes. Genetic summary statistics of POAG from the International Glaucoma Genetics Consortium, Global Biobank Meta‐analysis Initiative, and FinnGen consortia were analyzed for Europeans (38,164 cases and 1,576,179 controls) and East Asians (16,650 cases and 288,833 controls) separately. Inverse‐variance weighted random‐effects models were used as primary method.

**Results:**

MR results provided consistent evidence of a protective effect of ABCC8 inhibition on POAG using data sets from IGG, GBMI, and FinnGen. Genetically predicted one‐standard deviation reduction in HbA1c from ABCC8 inhibition were significant associated with lower risk of POAG in Europeans (OR = 0.211, 95% CI: 0.133–0.333; *p* < 0.001) and East Asians (OR = 0.070, 95% CI: 0.011–0.459; *p* = 0.0056). The association between genetically predicted ABCC8 inhibition and risk of POAG was mainly mediated through intraocular pressure. No association was found for PPARG, SLC5A2, or GLP1R. Sensitivity analyses supported this observation.

**Conclusions:**

We found a protective effect of genetically proxied ABCC8 inhibition on POAG risk in both Europeans and East Asians, highlighting ABCC8 as a promising candidate drug target for POAG, and mechanisms underlying the protective effect should also be investigated.

**Abbreviations**
ABCC8: ATP binding cassette subfamily C member 8
ALT: alanine aminotransferase
AST: aspartate aminotransferase
BBJ: BioBank Japan
BMI: body mass index
CI: confidence interval
DIAGRAM: DIAbetes Genetics Replication And Meta‐analysis
DPP‐4: dipeptidyl peptidase‐4
GBMI: Global Biobank Meta‐analysis Initiative
GLP1R: glucagon‐like peptide 1 receptor
GWAS: genome‐wide association study
IGGC: International Glaucoma Genetics Consortium
IOP: intraocular pressure
IVW: inverse variance‐weighted
MR: Mendelian randomization
OR: odds ratio
POAG: primary open‐angle glaucoma
PPARG: peroxisome proliferator‐activated receptor γ
PRESSO: Pleiotropy RESidual Sum and Outlier
RCT: randomized clinical trial
RNFL: retinal nerve fiber layer
SD: standard deviation
SGLT2: sodium‐glucose co‐transporter 2
SLC5A2: Solute Carrier Family 5 Member 2
SNP: single‐nucleotide polymorphism
T2D: type 2 diabetes
UKBB: UK Biobank

## 1 Introduction

Primary open‐angle glaucoma (POAG) characterized by progressive optic nerve degeneration and vision loss is the leading cause of irreversible blindness worldwide and is predicted to affect 80 million people by 2040 [1]. Although the relationship between older age, ethnicity, family history, type 2 diabetes (T2D) [2, 3, 4, 5, 6], elevated intraocular pressure (IOP) and POAG has been extensively investigated, IOP is the only well‐established modifiable risk factor, and all existing interventions focus on lowering IOP [7]. Most importantly, pharmaceutical agents commonly used for POAG treatment (e.g., prostaglandin analogs, carbonic anhydrase inhibitors, β blockers, etc.) have unwanted side effects and only slow the disease progression [7]. Thus, there is considerable interest in identifying new agents for improved interventions of POAG.

There are accumulative evidence reporting the protective effect of antidiabetic drug medication against POAG risk. For example, some observational studies have reported that metformin users have lower risk of POAG [8, 9, 10]. However, other studies suggest no beneficial effects of metformin usage in the reduction of POAG risk or delay progression of the disease [11, 12]. In addition, recent reports found a lower risk of POAG with glucagon‐like peptide‐1 receptor (GLP‐1R) agonists [13, 14], while another study suggests that T2D patients treated with sodium‐glucose co‐transporter 2 (SGLT2) inhibitors have a reduced risk of incident glaucoma, compared to GLP‐1R agonists [15]. There is also some evidence that sulfonylureas may decrease POAG risk owning to its neuroprotective effects in human and rodent retina [16]. By contrast epidemiological failed to demonstrate that sulfonylureas use could reduce the risk of POAG [10]. These epidemiological studies are vulnerable to selection bias (e.g., health‐insured populations, diabetic populations), unmeasured confounding, immortal time bias, and the causal relationship between antidiabetic medication and protective effect against POAG has not been determined. Meanwhile, no evidence is available from large‐scale randomized controlled trials (RCTs) on the protective effects of antidiabetics drugs against POAG.

Using germline variants within the genes encoding drug targets as genetic instruments (proxies) that mimic the effect of pharmacological agents, drug target Mendelian randomization (MR) is an efficient way to evaluate the causality for lifelong pharmacological exposures on disease outcomes. Since germline genetic variants are randomly assorted during meiosis and fixed at conception, MR avoids the bias of reverse causality and confounding factors [16]. Given the inconsistent reports of associations of different antidiabetic treatments with POAG risk in observational studies, we used drug target MR to simulate long‐term exposure to five approved T2D drugs with known mechanisms of action (sulfonylurea receptor 1 (ATP binding cassette subfamily C member 8 [ABCC8], peroxisome proliferator‐activated receptor γ [PPARG], dipeptidyl peptidase‐4 [DPP‐4], SGLT2, and GLP1R) in European and East Asian populations separately, to assess their causal effects on POAG.

## 2 Methods

### 2.1 Study Overview

**Figure 1:**
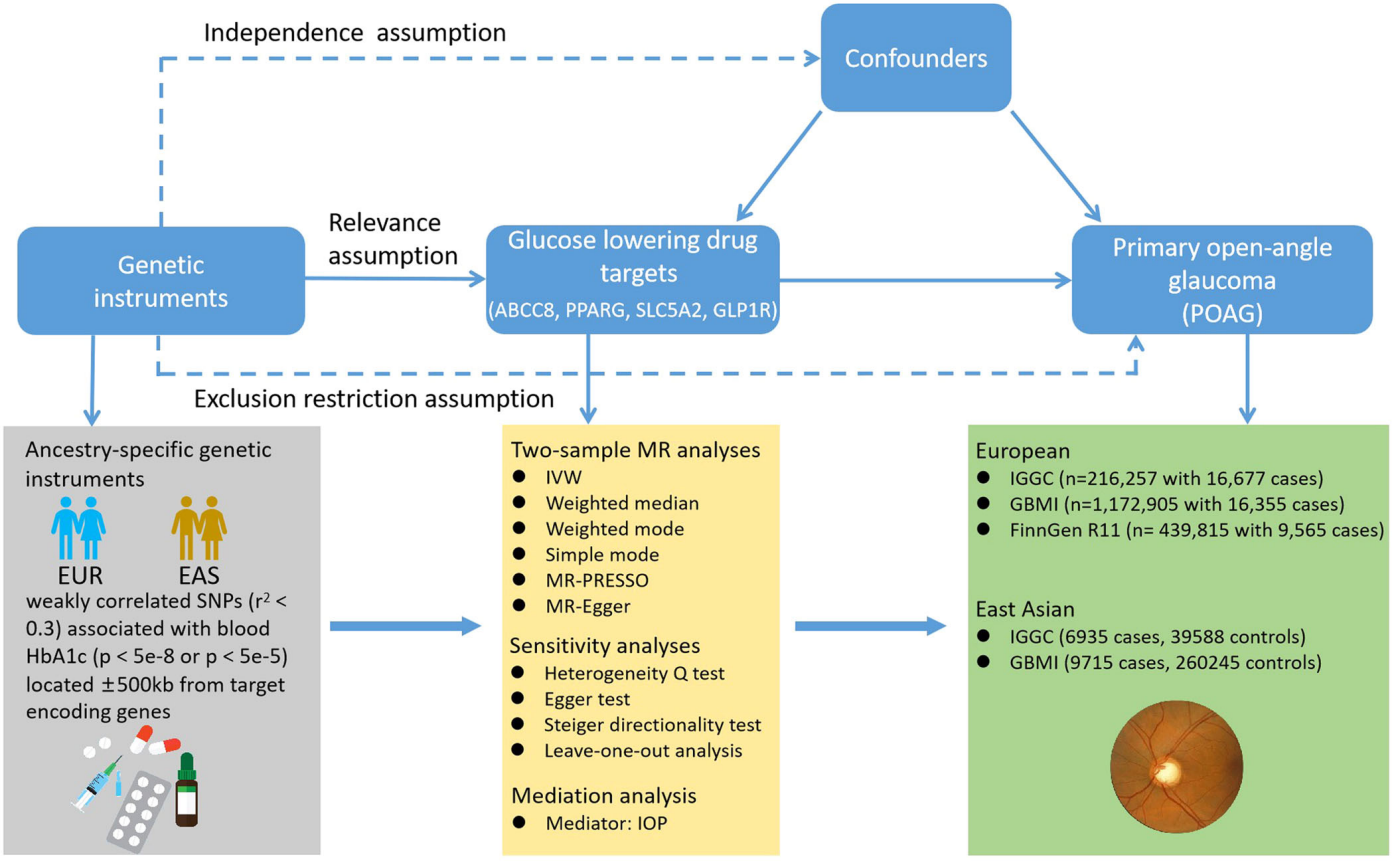
Outline of the study design. BCC8 = ATP binding cassette subfamily C member 8; GBMI = Global Biobank Meta‐analysis Initiative; GLP1R = glucagon‐like peptide 1 receptor; IGGC = International Glaucoma Genetics Consortium; IOP = intraocular pressure; IVW = inverse variance‐weighted; MR = Mendelian randomization; PPARG = peroxisome proliferator‐activated receptor γ; PRESSO = Pleiotropy RESidual Sum and Outlier; SLC5A2 = Solute Carrier Family 5 Member 2; SNP = single‐nucleotide polymorphism.

This study followed the Strengthening the Reporting of Observational Studies in Epidemiology‐MR reporting guidelines (Supporting Information S1: Table S1). An outline of the present multi‐ancestry two‐sample drug‐target MR study design is shown in Figure 1. First, we selected two sets of genetic instruments that were proxies of the effect of four antidiabetic drugs for Europeans and East Asians separately. Validity of the two set instruments were then checked using different positive control outcomes. Second, we assessed the effects of genetically proxied antidiabetic drugs on POAG risk in Europeans and East Asians using the established genetic instruments respectively. Detailed information on included datasets is summarized in Supporting Information S1: Table S2. Participant consent and ethical approval were not required for this study because all summary‐level genetic data were obtained from sources available to the public.

### 2.2 Instrument Construction

To generate genetic instruments to proxy antidiabetic drug target inhibition for Europeans, summary genetic association data were obtained from genome‐wide association study (GWAS) of glycated haemoglobin HbA1c levels in UK Biobank (*N* = 389,889 Europeans) [17]. Genetic instruments were constructed using the single‐nucleotide polymorphisms (SNPs) associated with HbA1c at genome‐wide significance (*p* < 5 × 10^−8^) that were in or within ± 500 kb from the gene encoding each respective target (PPARG, chr3:12328984‐12475855; ABCC8, chr11:17414432‐17498449; GLP1R, chr6:39016557‐39055520; SLC5A2, chr16:31494323‐31502181) as per GRCh37 assembly [18]. No genome‐wide significant SNPs within 500 kb windows from DPP4 (instruments for DPP‐4 inhibitors) were available and this target was excluded for MR analyses. Metformin targets were also excluded as it is mechanism(s) remain uncertain [19] and metformin use cannot be instrumented for MR [20]. For each of these drug targets, SNPs used as instruments were permitted to be in weak linkage disequilibrium (LD, *r* ^2^ < 0.30) with each other to increase the proportion of variance in each respective drug target explained by the instrument, maximising instrument strength [21]. To obtain more available SNPs for East Asian, independent (LD, *r* ^2^ < 0.30) SNPs associated with HbA1c were selected using a less stringent threshold of *p* < 5 × 10^−5^, within ± 500 kb regions of these target genes from BioBank Japan project (BBJ) consortium (*N* = 71,221 East Asians) [22, 23]. For both ancestries, the strength of genetic instruments was measured by calculating the proportion of variance of each drug target explained by the instrument (*r* ^2^) and *F* statistics [24]. As a convention, an *F* statistic of at least 10 indicates minimal weak instrument bias [25].

### 2.3 Instrument Validation

To validate the genetically proxied drug targets instruments, we initially assess the causal effects of the genetically predicted HbA1c level on T2D using the largetst GWAS meta‐analysis datasets [26]. Then, we examined their association with endpoints influenced by these medications in RCTs. Positive control analyses were performed with alanine aminotransferase (ALT) and aspartate aminotransferase (AST) levels as the outcomes for PPARG (i.e., PPARG agonists lower levels of ALT and AST) [27]. Likewise, we validated ABCC8 and GLP1R instruments by examining the association between inhibition of these targets and body mass index (BMI) as reported in clinical trials (i.e., sulfonylureas cause weight gain and GLP1R agonists cause weight loss) [28, 29]. Information regarding the GWAS datasets of T2D, ALT, AST, and BMI [23, 30] used in validation analyses for Europeans and East Asians were displayed in Supporting Information S1: Table S2.

### 2.4 Summary Level Genetic Data on POAG

In Europeans, GWAS summary results for POAG obtained from the International Glaucoma Genetics Consortium (IGGC) including 16,677 patients and 199,580 controls [31] were used during the discovery stage. For replication analyses, summary genetic association data were obtained on 16,355 POAG cases and 1,156,550 controls of European ancestry from the Global Biobank Meta‐analysis Initiative (GBMI) [32]. Because only part of FinnGen POAG data sets (4433 cases and 210,201 controls) were included in GBMI, we then used the data from the Finngen R11 release (9565 cases and 430,250 controls) [33] for additional replication analysis and thus minimize the possibility of overlapping between the exposure and outcome GWASs. Details of the included studies were described in Supporting Information S1: Table S2. Further information on diagnostic criteria for POAG, genotyping, imputation, and quality control measures for these studies is available in the original publications.

In East Asians, we used the largest GWAS data of POAG currently available from GBMI (9715 cases and 260,245 controls) as discovery data set and IGGC data set (6935 cases and 39,588 controls) for replication analysis (Supporting Information S1: Table S2).

### 2.5 Summary Level Genetic Data on IOP and Other Glaucoma Endophenotypes

We downloaded summary statistics for IOP from the UKBB of European participants. The GWAS results for Goldmann‐correlated IOP were available for left eyes (field identifier: 5263; *n* = 76,510) and right eyes (field identifier: 5255; *n* = 76,630) separately. GWAS summary data for glaucoma endophenotypes including retinal nerve fiber layer (RNFL) thickness [34], optic cup area [35], optic disc area [35], and vertical cup‐to‐disc ratio [36] were obtained from studies carried out in European descent (Supporting Information S1: Table S2).

## 3 Statistical Analysis

Causal estimates were generated using random‐effects inverse‐variance weighted (IVW) approach after adjustment of LD structure as the primary method [37]. The IVW effect estimate of drug proxies on POAG was calculated separately for IGGC, GBMI, and FinnGen, and then combined using random‐effects model. As unbiased IVW estimate depends on the absence of horizontal pleiotropy (or balanced horizontal pleiotropy) of included genetic instruments, we conducted sensitivity analyses which each make different assumptions using the MR‐Egger, weighted median [38], simple mode, and weighted mode [39]. If an SNP was missing from the outcome GWAS, we replaced it with a proxy SNP in high LD (*r* ^2^ > 0.80) using LDlink (https://ldlink.nci.nih.gov/). Potential horizontal pleiotropy was examined with the MR‐Egger intercept test, where deviation from zero denotes the presence of directional pleiotropy [40]. The presence of pleiotropy was also assessed using the Mendelian Randomization Pleiotropy RESidual Sum and Outlier (MR‐PRESSO) global test [41]. The heterogeneity between SNPs were evaluated by Cochran's *Q* statistic. If heterogeneity (where *Q* statistic *p* < 0.05) or pleiotropy were detected, the effect estimates are reassessed after outlier removal. To further clarify the impact of horizontal pleiotropy on causality estimates, we also examined the selected genetic instruments and their proxies (*r* ^2^ > 0.8) and their associations with potential confounders (*p* < 5 × 10^−8^) in Phenoscanner [42]. In addition, the MR Steiger test was performed to validate direction of association between antidiabetic drug target inhibition and POAG [43]. Next, iterative leave‐one‐out analysis was conducted by removing one SNP at a time from genetic instruments to assess the influence of individual SNP on the observed associations. The effect size was scaled to per standard deviation (SD) changes in HbA1c level.

To determine whether the observed association between drug targets and POAG was a direct association, we assessed the relationship between genetically proxied antidiabetic medication and IOP which is the only known modifiable risk factor for POAG. For significant associations, the exposure‐mediator‐outcome pathway may exist. We calculated the direct effect of genetically proxied antidiabetic therapies on POAG risks using the “Two‐Step MR” method [44]. The indirect effects of genetically proxied antidiabetic therapies on POAG risk via IOP were assessed with the “Product of coefficients” method [45] and the total effect were generated using univariable analyses. Standard errors for the indirect effects and the proportion mediated were estimated using bootstrapping.

To account for multiple testing across analyses, a Bonferroni correction was used to establish a *p* value threshold of < 0.0125 (0.05/4, four drug targets tested). Type I error rate was set at 0.05 for two‐sided analysis. We calculated the statistical power of our analyses using an online tool (http://cnsgenomics.com/shiny/mRnd/) [46]. All the analyses were conducted using the TwoSampleMR (version 0.5.7), MendelianRandomization (version 0.9.0), meta (version 6.5‐0), and TwoStepCisMR (version 0.0.0.9000) packages in R platform (version 4.3.0).

## 4 Results

### 4.1 Genetic Variants Selection

Characteristics of genetic variants used to instrument antidiabetic drug targets are presented in Supporting Information S1: Tables S3–S7. In total, 6 SNPs in ABCC8 (5 for Europeans and 1 for East Asians) were used to proxy ABCC8 inhibition; 5 SNPs in GLP1R (4 for Europeans and 1 for East Asians) proxied GLP1R agonists; 34 SNPs in PPARG (for Europeans only) proxied PPARG agonists and 11 SNPs in SLC5A2 (for Europeans only) proxied SGLT2 inhibitors. Across all these drug targets, F statistics for their respective instruments are greater than 10 (Supporting Information S1: Table S3), suggesting that weak instrument bias was unlikely to affect the conclusions.

### 4.2 Instrument Validation

The positive control analyses identified a MR association in the expected direction between genetically proxied drug targets and T2D (Supporting Information S1: Table S8). In addition, genetically proxied ABCC8 inhibition was also associated with elevated BMI for Europeans (odds ratio [OR] = 1.749, 95% confidence interval [CI]: 1.502–2.038; *p* < 0.001), while targeting GLP1R might reduce such a risk (OR = 0.810, 95% CI: 0.665–0.986; *p* 0.036). Genetically proxied PPARG inhibition was associated with lower levels of ALT (OR = 0.669, 95% CI: 0.623–0.718) and AST (OR = 0.710, 95% CI: 0.657–0.766) in Europeans. We also found similar results for the association between genetically predicted ABCC8 and GLP1R inhibition and BMI for East Asians (Supporting Information S1: Table S8).

### 4.3 MR Estimates for POAG in Europeans

**Figure 2:**
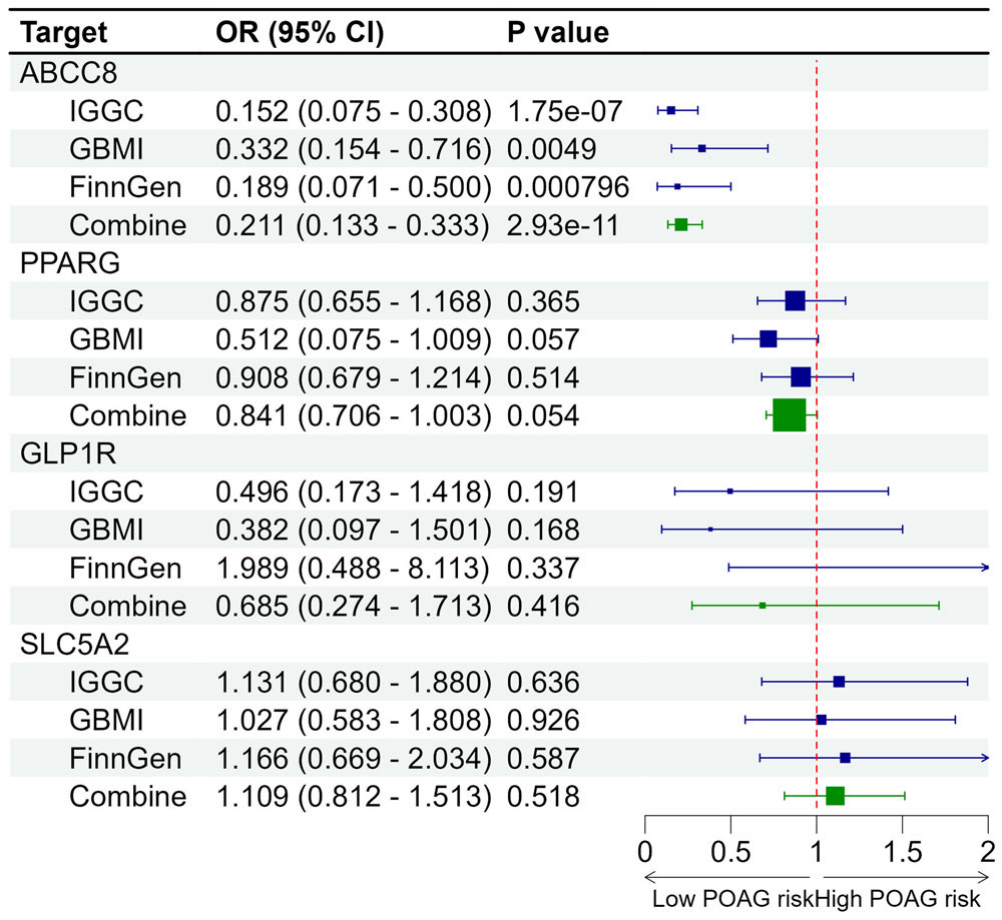
Causal effect of genetically proxied antidiabetic drug targets on POAG in Europeans. MR was estimated by generalized inverse variance weighted method. Data are presented as the change of primary open‐angle glaucoma risk for 1 − SD reduction of HbA1c.

Genetically proxied ABCC8 inhibition showed strong evidence of association with decreased POAG risk using data from IGGC in the discovery stage with an OR of 0.152 (95% CI: 0.075–0.308; *p* < 0.001, per SD reduction in HbA1c). The main analysis was also supported by the analysis using data from GBMI (OR = 0.332, 95% CI: 0.154–0.716; *p* = 0.0049) and FinnGen (OR = 0.189, 95% CI: 0.071–0.500; *p* < 0.001, Supporting Information S1: Figure S1). MR Findings from 1 SD reduction in HbA1c by targeting ABCC8 were associated with lower risk of POAG with a pooled OR of 0.211 (95% CI = 0.133–0.333, *p* < 0.001, Figure 2). Sensitivity analyses using the MR‐Egger, weighted median, weighted mode, and simple mode provided consistent association (Supporting Information S1: Table S9). The intercept from the MR‐Egger regression analysis and heterogeneity from Cochran's *Q* test (all *p* > 0.05) implied little evidence of the existence of directional pleiotropy or heterogeneity (Supporting Information S1: Table S10). Furthermore, the MR‐PRESSO global test (*p* for global test > 0.05) did not detect any outlier SNPs in all datasets and the Steiger directionality test confirmed that ABCC8 inhibition have a causal effect on POAG (Supporting Information S1: Table S10). Given a type 1 error of 5%, we had sufficient power (> 80%) when the expected OR was < 0.20 for POAG (Supporting Information S1: Table S11). Moreover, these results were robust in the leave‐one‐out sensitivity analysis (Supporting Information S1: Figure S2).

We further explored genetic associations between ABCC8 and glaucoma‐related endophenotypes, including IOP, RNFL thickness, optic cup area, optic disc area, and vertical cup‐to‐disc ratio using data sets from European ancestry (Supporting Information S1: Table S12). Genetically proxied ABCC8 inhibition was found associated with IOP (*p* < 0.001) and RNFL thickness (*p* < 0.001), but not associated with any of the other glaucoma endophenotypes.

There was no MR evidence of association of genetically proxied PPARG, GLP1R, or SLC5A2 inhibition with POAG risk (Figure 2, Supporting Information S1: Table S9).

### 4.4 MR Estimates for POAG in East Asians

**Figure 3:**
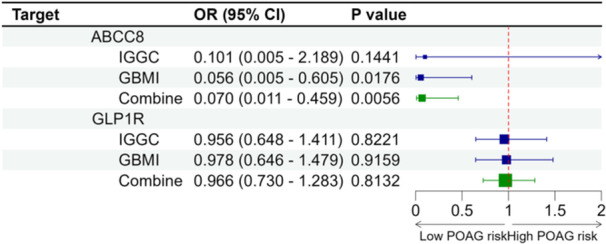
Causal effect of genetically proxied antidiabetic drug targets on POAG in East Asians. MR was estimated by generalized inverse variance weighted method. Data are presented as the change of primary open‐angle glaucoma risk for 1 − SD reduction of HbA1c.

The genetic mimicry of ABCC8 inhibition also presented protective effects on POAG in East Asians with combined OR of 0.070 (95% CI: 0.011–0.459, *p* = 0.0056, per SD reduction in HbA1c, Figure 3). The correct causal direction was confirmed using the Steiger directionality test (Supporting Information S1: Table S10). No significant associations between genetically proxied GLP1R inhibition and POAG risk were detected (Figure 3, Supporting Information S1: Table S13).

### 4.5 Mediation Analysis

**Figure 4:**
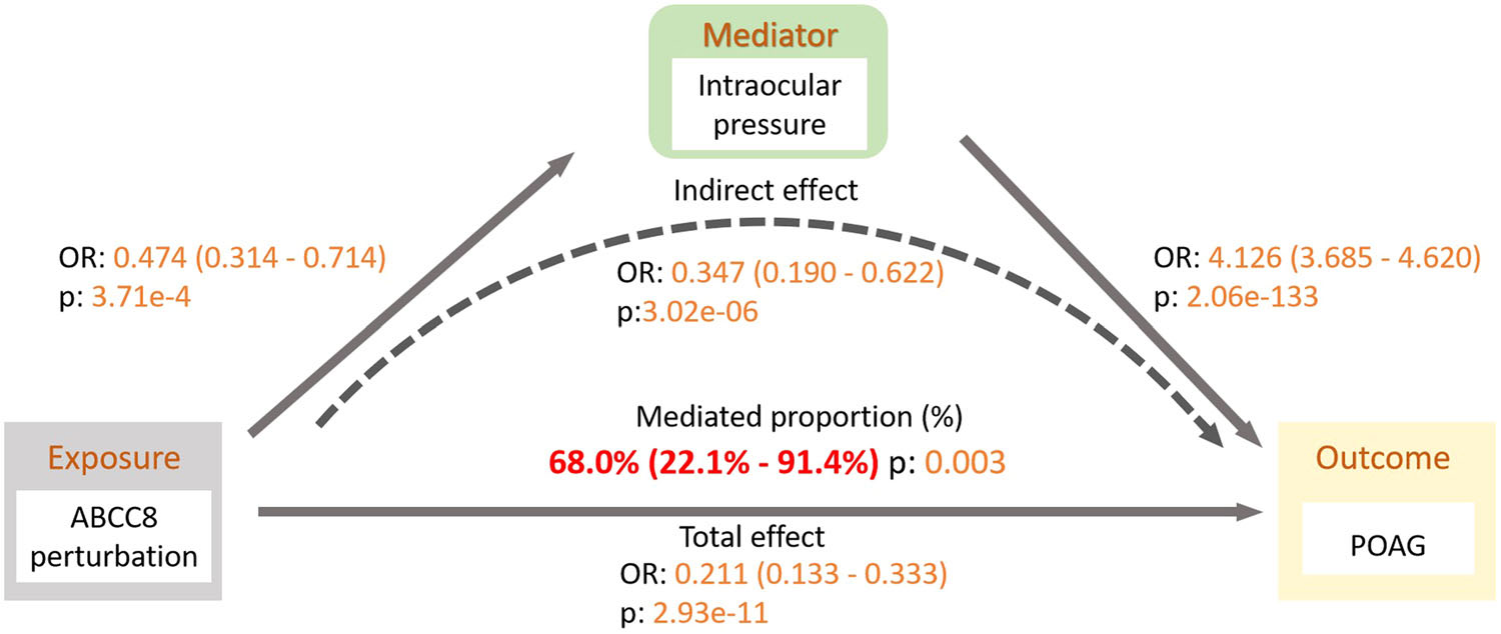
Mediation analysis of the effect of genetically proxied ABCC8 inhibition on POAG via intraocular pressure under a two‐step Mendelian randomisation analysis framework. “Direct effect” indicates the effect of genetically proxied ABCC8 inhibition on POAG risk. “Indirect effect” indicates the effect of genetically proxied ABCC8 inhibition on POAG risk through the intraocular pressure.

Given that IOP is the only well‐established modifiable risk factor for POAG, we then performed two‐step MR analysis to investigate the indirect effect of ABCC8 inhibition on POAG. MR analyses provided strong evidence for genetically proxied IOP and POAG (OR = 4.126, 95% CI: 3.685–4.620; *p* < 0.001, Supporting Information S1: Table S14). Using IOP data from UKBB, we found genetically proxied ABCC8 inhibition were significantly associated with decreased IOP (OR = 0.474, 95% CI: 0.314–0.714; *p* < 0.001, Supporting Information S1: Table S15). The association between genetically predicted ABCC8 inhibition and decreased risk of POAG was mediated through IOP (mediation proportion: 68.0%, *p* = 0.003, Figure 4).

## 5 Discussion

In the present large‐scale MR study, using the currently available largest genetic data for HbA1c and POAG, we found strong evidence of the association of genetically proxied ABCC8 inhibition with decreased risk of POAG. Importantly, the association is consistent between studies in European ancestry and studies in East Asian ancestry. Our study provided strong evidence that ABCC8 is a promising drug target for POAG and the protective effect is likely to be due to a mechanism involving the lowering of IOP.

MR analyses provided similar central effect estimate and overlapped CIs for genetically proxied ABCC8 estimates of POAG in Europeans and East Asians. However, the observed association among East Asians was based on a smaller set of genetic instruments available for ABCC8 target (1 SNP) and the robustness of our IVW estimates cannot be interrogated by sensitivity analysis. Therefore, investigations should be repeated in the future when more genetic instruments are available from larger HbA1c GWAS of East Asians.

ABCC8 is known as one of the regulatory sulfonylurea subunits of ATP‐sensitive potassium (K_ATP_) channels, although thus far its role in IOP has yet to be comprehensively evaluated and characterized. Preclinical study have identified functional K_ATP_ channels in human eyes and active K_ATP_ channels (e.g., diazoxide, nicorandil, or cromakalim treatment) increase outflow facility thus lowered pressure in human anterior segment organ culture and in rat eyes [47, 48]. Recently, water soluble analogs of the K_ATP_ channel opener cromakalim have shown IOP‐lowering effect of the desired magnitude in mouse and rabbit model without toxic effects on cell structure or alterations to the aqueous humor outflow pathway [49]. Likewise, our mediation analysis revealed that protective effect of genetically proxied ABCC8 inhibition on decreased POAG risk was partially mediated by lower IOP. Furthermore, K_ATP_ channels have been proved as key molecules to provide retinal neuroprotection [50]. We also found a significant association between genetically proxied ABCC8 inhibition and RNFL thickness in many of the MR models tested. Our findings add to growing evidence suggesting that K_ATP_ channel openers may provide the most benefit toward reducing POAG risk with both hypotensive and neuroprotective properties.

Despite the association between T2D and an increased risk of POAG, the underlying mechanism is not fully understood. Although we observed an association of genetically proxied ABCC8 inhibition on the outcome, it is not known whether ABCC8 agonists directly alter POAG risk independent of their impact on blood glucose levels and the clinical effects of these medications on nondiabetic populations or patients with normal IOP remain unknown. The missense variant rs757110 (included in the genetic instruments for ABCC8) was associated with a response to sulfonylureas by interacting with A‐site of SUR1 [51, 52]. Observation study found that this SNP significantly associated with percent decrease of fasting plasma glucose among T2D patients after treatment [53]. Another functional study reported that rs757110 affected the splicing efficiency of exon 33, suggesting that this SNP may play a modifier role in ABCC8‐related disorders or drug response [54]. Therefore, detailed mechanisms linking ABCC8 and POAG need to be investigated in the future.

Regarding the protective effect of antidiabetic drugs (e.g, metformin and GLP‐1R agonists) on POAG, prior observational studies have generated conflicting results, and as of yet, no direct correlation between antidiabetic medications and glaucoma endophenotypes (e.g., IOP, central corneal thickness, cup‐disc ratio, or visual field) has been established [8, 9, 10, 11, 12, 13, 14]. Interpretation of results from pharmacoepidemiological studies remain challenging because confounding by indication and confounding by the environmental and lifestyle factors of patients cannot be fully adjusted for using observational studies. The interpretation can be even more complicated due to inappropriate control groups, the inclusion of “prevalent users” of medications, short intervention or follow‐up periods, and medication compliance. Although there was little evidence to support an association of genetically proxied ABCC8 inhibition with POAG from observational studies, the present MR study is unlikely to be affected by confounders and directly evaluate the causal effect of ABCC8 inhibition on POAG risk.

Our study has number of important strengths. To our knowledge, this is the first genetic instrumental variable study to explore the long‐term effect of antidiabetic drugs on POAG in diverse ethnic populations (Europeans and East Asians). We used cis‐acting variants with *F* statistics > 10 to mimic antidiabetic drugs of interest, and therefore minimized horizontal pleiotropy. In addition, positive control analysis with T2D and established secondary effects of medications were used to validate the selected instruments thus enhance robustness of the results. Furthermore, we leveraged large‐scale summary statistics available from several GWAS consortia, which greatly enhanced the statistical power and precision of causal estimates.

Despite the novelty of the study, several limitations should be acknowledged. First, the relationship between ABCC8 and POAG was based on genetic proxies of therapeutic targets that may not be equivalent to the short‐term effects of those antidiabetic drugs. This is because drugs are often taken for a defined period, whereas germline genetic variants are conventionally interpreted as lifelong exposures to risk factors of interest. Thus, our study is more helpful in assessing the direction of associations rather than quantifying the magnitude of causal effect. Second, although we performed various sensitivity analyses and the causal associations were estimated by different MR approaches, we cannot definitively rule out the possibility that instruments used in the MR associated with the risk of POAG through a pleiotropic pathway. Third, we were unable to evaluate the causal effect of other commonly used antidiabetic drug targets (e.g., metformin, and DPP‐4 inhibitors) due to the absence of reliable genetic instruments. Fourth, given that over 50% of glaucoma is not diagnosed until irreversible optic nerve damage has occurred [1], we cannot rule out the possibility that controls in POAG GWAS included individuals with latent, undiagnosed cases, the presence of which would bias associations toward or away from the null. Fifth, gene‐environment and/or drug–drug interactions may bias effect estimates as a growing amount of evidence suggested that associations between genetic instruments and exposures may vary throughout the life course [55, 56]. Sixth, the number of available genetic instruments among East Asians was relatively small, further confirmation in larger studies of East Asians is warranted. Finally, our study only predicts on‐target effects of antidiabetic medications on POAG, whereas the potential off‐target effects cannot be estimated.

## 6 Conclusions

In summary, we developed solid instruments for PPARG, ABCC8, and GLP1R using strict validation methods and found MR evidence between genetic proxies of ABCC8 inhibition and reduced POAG risk. Moreover, this study suggested that ABCC8 is a promising candidate drug target for POAG and part of the mechanism may be involving the lowering of IOP. Future studies should investigate the role of ABCC8 and glaucoma endophenotypes in POAG susceptibility. Additional clinical studies are warranted in repurposing or retargeting sulfonylurea as a prevention or treatment POAG.

## Author Contributions

**Kefu Tang:** conceptualization; investigation; writing–original draft; writing–review and editing; visualization; validation; methodology; software; formal analysis; project administration; resources; supervision; data curation. **Wenqiu Wang:** conceptualization; writing–review and editing; software; methodology; resources; validation. **Weiteng Chang:** conceptualization; writing–review and editing; methodology; software; resources; validation. **Xi Wu:** investigation; methodology; visualization; writing–review and editing; project administration; formal analysis; software; data curation; supervision; resources.

## Ethics Statement

Human subjects were not included in this study. Participant consent and ethical approval were not required for this study because all summary‐level genetic data were obtained from sources available to the public.

## Conflicts of Interest

The authors declare no conflicts of interest.

## Transparency Statement

The lead author Kefu Tang affirms that this manuscript is an honest, accurate, and transparent account of the study being reported; that no important aspects of the study have been omitted; and that any discrepancies from the study as planned (and, if relevant, registered) have been explained.

## Supporting information

Supporting information.

## Data Availability

All authors have read and approved the final version of the manuscript. Kefu Tang had full access to all of the data in this study and takes complete responsibility for the integrity of the data and the accuracy of the data analysis. Detailed information for these publicly available datasets used in the study is summarized in Supporting Information S1: Table S2.
