# Supplementary material for: Genetically Proxied Antidiabetic Drug Target and Primary Open‐Angle Glaucoma: A Mendelian Randomization Study
Source: Health Sci Rep. 2024 Oct 24;7(10):e70162. doi: 10.1002/hsr2.70162 (PMC11499708; doi:10.1002/hsr2.70162)
Supplement: Supplementary file 1 — Supporting information. [file HSR2-7-e70162-s001.docx]

**Table S1. STROBE-MR checklist of recommended items to address in reports of Mendelian randomization studies**

| **Item No.** | **Section** | **Checklist item** | **Section** |
| --- | --- | --- | --- |
| 1 | **TITLE and ABSTRACT** | Indicate Mendelian randomization (MR) as the study’s design in the title and/or the abstract if that is a main purpose of the study | Title page & Abstract page |
|  | **INTRODUCTION** |  |  |
| 2 | **Background** | Explain the scientific background and rationale for the reported study. What is the exposure? Is a potential causal relationship between exposure and outcome plausible? Justify why MR is a helpful method to address the study question | Introduction (paragraphs 1-2) |
| 3 | **Objectives** | State specific objectives clearly, including pre-specified causal hypotheses (if any). State that MR is a method that, under specific assumptions, intends to estimate causal effects | Introduction (paragraphs 3) |
|  | **METHODS** |  |  |
| 4 | **Study design and data sources** | Present key elements of the study design early in the article. Consider including a table listing sources of data for all phases of the study. For each data source contributing to the analysis, describe the following: | Method (Study Overview section) |
|  | a) | Setting: Describe the study design and the underlying population, if possible. Describe the setting, locations, and relevant dates, including periods of recruitment, exposure, follow-up, and data collection, when available. | Method (Instrument construction section & Summary level genetic data on POAG section) , Table S2 |
|  | b) | Participants: Give the eligibility criteria, and the sources and methods of selection of participants. Report the sample size, and whether any power or sample size calculations were carried out prior to the main analysis | Method (Study Overview section), Table S2 |
|  | c) | Describe measurement, quality control and selection of genetic variants | Method (Study Overview section), Table S2 |
|  | d) | For each exposure, outcome, and other relevant variables, describe methods of assessment and diagnostic criteria for diseases | Method (Instrument construction section & Summary level genetic data on POAG section) , Table S2 |
|  | e) | Provide details of ethics committee approval and participant informed consent, if relevant | Method (Study Overview section) |
| 5 | **Assumptions** | Explicitly state the three core IV assumptions for the main analysis (relevance, independence and exclusion restriction) as well assumptions for any additional or sensitivity analysis | Method (Instrument construction) |
| 6 | **Statistical methods: main analysis** | Describe statistical methods and statistics used | Method (Statistical Analysis paragraph 1-3) |
|  | a) | Describe how quantitative variables were handled in the analyses (i.e., scale, units, model) | Method (Statistical Analysis paragraph 1) |
|  | b) | Describe how genetic variants were handled in the analyses and, if applicable, how their weights were selected | Method (Statistical Analysis paragraph 1) |
|  | c) | Describe the MR estimator (e.g. two-stage least squares, Wald ratio) and related statistics. Detail the included covariates and, in case of two-sample MR, whether the same covariate set was used for adjustment in the two samples | Method (Statistical Analysis paragraph 1) |
|  | d) | Explain how missing data were addressed | N/A |
|  | e) | If applicable, indicate how multiple testing was addressed | Method (Statistical Analysis paragraph 3) |
| 7 | **Assessment of assumptions** | Describe any methods or prior knowledge used to assess the assumptions or justify their validity | Method (Instrument validation) |
| 8 | **Sensitivity analyses and additional analyses** | Describe any sensitivity analyses or additional analyses performed (e.g. comparison of effect estimates from different approaches, independent replication, bias analytic techniques, validation of instruments, simulations) | Method (Statistical Analysis paragraph 1-3) |
| 9 | **Software and pre-registration** |  |  |
|  | a) | Name statistical software and package(s), including version and settings used | Method (Statistical Analysis paragraph 3) |
|  | b) | State whether the study protocol and details were pre-registered (as well as when and where) | N/A |
|  | **RESULTS** |  |  |
| 10 | **Descriptive data** |  |  |
|  | a) | Report the numbers of individuals at each stage of included studies and reasons for exclusion. Consider use of a flow diagram | Figure 1 |
|  | b) | Report summary statistics for phenotypic exposure(s), outcome(s), and other relevant variables (e.g. means, SDs, proportions) | Table S2 |
|  | c) | If the data sources include meta-analyses of previous studies, provide the assessments of heterogeneity across these studies | N/A |
|  | d) | For two-sample MR:  i.  Provide justification of the similarity of the genetic variant-exposure associations between the exposure and outcome samples  ii.  Provide information on the number of individuals who overlap between the exposure and outcome studies | Method (Summary level genetic data on POAG paragraph 3) |
| 11 | **Main results** |  |  |
|  | a) | Report the associations between genetic variant and exposure, and between genetic variant and outcome, preferably on an interpretable scale | Results (MR estimates for POAG in Europeans & MR estimates for POAG in East Asians) |
|  | b) | Report MR estimates of the relationship between exposure and outcome, and the measures of uncertainty from the MR analysis, on an interpretable scale, such as odds ratio or relative risk per SD difference | Results (MR estimates for POAG in Europeans & MR estimates for POAG in East Asians) |
|  | c) | If relevant, consider translating estimates of relative risk into absolute risk for a meaningful time period | N/A |
|  | d) | Consider plots to visualize results (e.g. forest plot, scatterplot of associations between genetic variants and outcome versus between genetic variants and exposure) | Figure 2-3 |
| 12 | **Assessment of assumptions** |  |  |
|  | a) | Report the assessment of the validity of the assumptions | Results (Mediation analysis) |
|  | b) | Report any additional statistics (e.g., assessments of heterogeneity across genetic variants, such as *I^2^*, Q statistic or E-value) | Table S10 |
| 13 | **Sensitivity analyses and additional analyses** |  |  |
|  | a) | Report any sensitivity analyses to assess the robustness of the main results to violations of the assumptions | Table S9, Table S10 |
|  | b) | Report results from other sensitivity analyses or additional analyses | Table S9, Figure S1. |
|  | c) | Report any assessment of direction of causal relationship (e.g., bidirectional MR) | Table S10 |
|  | d) | When relevant, report and compare with estimates from non-MR analyses | N/A |
|  | e) | Consider additional plots to visualize results (e.g., leave-one-out analyses) | Figure S2 |
|  | **DISCUSSION** |  |  |
| 14 | **Key results** | Summarize key results with reference to study objectives | Discussion (paragraphs 1) |
| 15 | **Limitations** | Discuss limitations of the study, taking into account the validity of the IV assumptions, other sources of potential bias, and imprecision. Discuss both direction and magnitude of any potential bias and any efforts to address them | Discussion (paragraphs 7) |
| 16 | **Interpretation** |  |  |
|  | a) | Meaning: Give a cautious overall interpretation of results in the context of their limitations and in comparison with other studies | Discussion (paragraphs 4) |
|  | b) | Mechanism: Discuss underlying biological mechanisms that could drive a potential causal relationship between the investigated exposure and the outcome, and whether the gene-environment equivalence assumption is reasonable. Use causal language carefully, clarifying that IV estimates may provide causal effects only under certain assumptions | Discussion (paragraphs 3) |
|  | c) | Clinical relevance: Discuss whether the results have clinical or public policy relevance, and to what extent they inform effect sizes of possible interventions | Discussion (paragraphs 8) |
| 17 | **Generalizability** | Discuss the generalizability of the study results (a) to other populations, (b) across other exposure periods/timings, and (c) across other levels of exposure | Discussion (paragraphs 2) |
|  | **OTHER INFORMATION** |  |  |
| 18 | **Funding** | Describe sources of funding and the role of funders in the present study and, if applicable, sources of funding for the databases and original study or studies on which the present study is based | None. |
| 19 | **Data and data sharing** | Provide the data used to perform all analyses or report where and how the data can be accessed, and reference these sources in the article. Provide the statistical code needed to reproduce the results in the article, or report whether the code is publicly accessible and if so, where | Data availability statement & Table S2 |
| 20 | **Conflicts of Interest** | All authors should declare all potential conflicts of interest | The authors declare that they have no conflict of interest to disclose. |

Table S2. Phenotype descriptions and data source.

| **Phenotypes** | **Study / Consortium** | **First Author (Year)** | **PMID** | **Sample size** | **Cases** | **Controls** | **Ancestry** | **GWAS ID** | **Biobank involved** | **Data source** |
| --- | --- | --- | --- | --- | --- | --- | --- | --- | --- | --- |
| Glycated haemoglobin HbA1c levels |  | Mbatchou J, et al. 2021 | 34017140 | 389889 |  |  | European | ebi-a-GCST90014006 | UK Biobank (UKBB) | <https://gwas.mrcieu.ac.uk/> |
| Glycated haemoglobin HbA1c levels |  | Sakaue S, et al. 2021 | 34594039 | 179000 |  |  | East Asian |  | BioBank Japan (BBJ) | <https://biobankjp.org/en/index.html> |
| POAG | IGGC | Gharahkhani P, et al. 2021 | 33627673 | 216257 | 16677 | 199580 | European | GCST90011766 |  | https://www.ebi.ac.uk/gwas/downloads/summary-statistics |
| POAG | GBMI | Zhou W, et al. 2022 | 36777996 | 1172905 | 16355 | 1156550 | European |  | Vanderbilt University Medical Center’s biobank (BioVU), Colorado Center for Personalized Medicine (CCPM), Iceland Biobank(DECODE), Estonian Biobank (ESTBB), Finnish biobank (FinnGen), Generation Scotland (GS), Trøndelag Health Study (HUNT), Lifelines Biobank (Lifelines), Mass General Brigham Biobank (MGBB), Michigan Genomics Initiative (MGI), The QSkin Sun and Health Study (Qskin), UK Biobank (UKBB) | https://www.globalbiobankmeta.org/resources |
| POAG | FinnGen (R11 release) | Kurki MI, et al. 2023 | 36653562 | 399805 | 9565 | 430250 | European |  | Finnish biobank (FinnGen) | https://www.finngen.fi/en |
| POAG | IGGC | Gharahkhani P, et al. 2021 | 33627673 | 46523 | 6935 | 39588 | East Asian | GCST90011768 |  | https://www.ebi.ac.uk/gwas/downloads/summary-statistics |
| POAG | GBMI | Zhou W, et al. 2022 | 36777996 | 269960 | 9715 | 260245 | East Asian |  | Biobank Japan (BBJ), Taiwan Biobank (TWB) | https://www.globalbiobankmeta.org/resources |
| T2D | DIAGRAM | Suzuki K, et al. 2024 | 38374256 | 1812013 | 242283 | 1569730 | European |  |  | http://diagram-consortium.org/ |
| T2D | DIAGRAM | Suzuki K, et al. 2024 | 38374256 | 427504 | 88109 | 339395 | East Asian |  |  | http://diagram-consortium.org/ |
| BMI | GIANT | Yengo L, et al. 2018 | 30124842 | 681275 |  |  | European | ieu-b-40 |  | <https://gwas.mrcieu.ac.uk/> |
| BMI |  | Sakaue S, et al. 2021 | 34594039 | 163835 |  |  | East Asian | ebi-a-GCST90018727 | BioBank Japan (BBJ) | <https://gwas.mrcieu.ac.uk/> |
| Alanine aminotransferase levels |  | Mbatchou J, et al. 2021 | 34017140 | 389733 |  |  | European | ebi-a-GCST90013992 | UK Biobank (UKBB) | [https://gwas.mrcieu.ac.uk/](https://gwas.mrcieu.ac.uk/) |
| Aspartate aminotransferase levels |  | Mbatchou J, et al. 2021 | 34017140 | 388490 |  |  | European | ebi-a-GCST90013996 | UK Biobank (UKBB) | <https://gwas.mrcieu.ac.uk/> |
| IOP, Goldmann-correlated (right eye) |  |  |  | 76630 |  |  | European |  | UK Biobank (UKBB) | http://www.nealelab.is/uk-biobank |
| IOP, Goldmann-correlated (left eye) |  |  |  | 76510 |  |  | European |  | UK Biobank (UKBB) | http://www.nealelab.is/uk-biobank |
| Optic cup area |  | Springelkamp H, et al. 2017 | 28073927 | 22484 |  |  | European | ebi-a-GCST004137 |  | <https://gwas.mrcieu.ac.uk/> |
| Optic disc area |  | Springelkamp H, et al. 2017 | 28073927 | 22504 |  |  | European | ebi-a-GCST004076 |  | <https://gwas.mrcieu.ac.uk/> |
| Vertical cup-to-disc ratio |  | Bonnemaijer PWM et al. 2019 | 31798171 | 25180 |  |  | European | GCST009412 |  | <https://www.ebi.ac.uk/gwas/downloads/summary-statistics> |
| Retinal nerve fiber layer thickness |  | Currant H, et al. 2021 | 33979322 | 31434 |  |  | European | GCST90014266 |  | <https://www.ebi.ac.uk/gwas/downloads/summary-statistics> |

Note: T2D = type 2 diabetes; POAG = primary open-angle glaucoma; IOP = intraocular pressure; BMI = body mass index; NA = not available; GBMI = Global Biobank Meta-analysis Initiative; IGGC = international Glaucoma Genetics Consortium; DIAGRAM = DIAbetes Genetics Replication And Meta-analysis; GIANT = Genetic Investigation of ANthropometric Traits

**Table S3. Characteristics of variants used as genetic instrument to proxy drug targets.**

| **Ancestry** | **Gene** | **SNP** | **Chr** | **Pos** | **Effect allele** | **Other allele** | **Effect allele frequency** | **Beta** | **SE** | **P value** | **F-statistics** |
| --- | --- | --- | --- | --- | --- | --- | --- | --- | --- | --- | --- |
| European | ABCC8 | rs10832777 | 11 | 17385229 | C | G | 0.42 | -0.0126352 | 0.00204281 | 6.20E-10 | 30.37 |
| European | ABCC8 | rs2883548 | 11 | 17377754 | G | A | 0.33 | -0.0145146 | 0.0021603 | 1.83E-11 | 36.57 |
| European | ABCC8 | rs61880293 | 11 | 17376498 | C | T | 0.06 | 0.020748 | 0.00378893 | 4.35E-08 | 17.84 |
| European | ABCC8 | rs664382 | 11 | 17206519 | T | A | 0.35 | 0.0120003 | 0.00215709 | 2.65E-08 | 25.52 |
| European | ABCC8 | rs757110 | 11 | 17418477 | A | C | 0.62 | -0.0219322 | 0.0021001 | 1.57E-25 | 88.30 |
| European | GLP1R | rs10305518 | 6 | 39055012 | G | T | 0.06 | 0.0277293 | 0.00437475 | 2.32E-10 | 36.15 |
| European | GLP1R | rs6904583 | 6 | 39358111 | T | C | 0.03 | 0.0368369 | 0.0063865 | 8.02E-09 | 27.61 |
| European | GLP1R | rs910166 | 6 | 39033168 | A | G | 0.08 | 0.0221889 | 0.00362703 | 9.50E-10 | 28.79 |
| European | GLP1R | rs9470964 | 6 | 39016096 | G | A | 0.06 | 0.0270651 | 0.0046939 | 8.12E-09 | 30.24 |
| European | PPARG | rs112496224 | 3 | 12301915 | T | C | 0.04 | -0.0429422 | 0.00523311 | 2.29E-16 | 67.34 |
| European | PPARG | rs115633989 | 3 | 12176274 | G | A | 0.05 | -0.033201 | 0.00458093 | 4.24E-13 | 52.53 |
| European | PPARG | rs116354045 | 3 | 11979131 | G | C | 0.02 | -0.0390164 | 0.00681545 | 1.04E-08 | 32.77 |
| European | PPARG | rs13059878 | 3 | 12500627 | C | T | 0.26 | -0.0190236 | 0.00227358 | 5.90E-17 | 70.01 |
| European | PPARG | rs13320580 | 3 | 11956181 | A | G | 0.46 | -0.0126085 | 0.00211463 | 2.48E-09 | 35.55 |
| European | PPARG | rs17036143 | 3 | 12294727 | T | G | 0.13 | 0.0239252 | 0.00298291 | 1.05E-15 | 64.33 |
| European | PPARG | rs17669026 | 3 | 12042835 | G | A | 0.16 | -0.0152739 | 0.00267922 | 1.19E-08 | 32.50 |
| European | PPARG | rs17669062 | 3 | 12043966 | C | T | 0.06 | -0.0261171 | 0.00375695 | 3.61E-12 | 48.33 |
| European | PPARG | rs17671592 | 3 | 12273414 | C | T | 0.05 | -0.0265086 | 0.00479321 | 3.19E-08 | 30.59 |
| European | PPARG | rs1822534 | 3 | 12266804 | G | A | 0.35 | -0.0407818 | 0.00206684 | 1.16E-86 | 389.33 |
| European | PPARG | rs2055740 | 3 | 12791009 | A | G | 0.96 | -0.0251026 | 0.00459701 | 4.74E-08 | 29.82 |
| European | PPARG | rs2305398 | 3 | 12856856 | G | A | 0.56 | -0.0153642 | 0.00207401 | 1.28E-13 | 54.88 |
| European | PPARG | rs2454429 | 3 | 12620720 | A | G | 0.50 | -0.0161446 | 0.00203931 | 2.44E-15 | 62.67 |
| European | PPARG | rs2600258 | 3 | 12004940 | G | A | 0.74 | -0.0142746 | 0.00247254 | 7.78E-09 | 33.33 |
| European | PPARG | rs2920500 | 3 | 12323413 | A | G | 0.56 | 0.0198787 | 0.00202645 | 1.02E-22 | 96.23 |
| European | PPARG | rs2921186 | 3 | 12385469 | T | C | 0.13 | 0.0257436 | 0.00286542 | 2.61E-19 | 80.72 |
| European | PPARG | rs307586 | 3 | 12110247 | T | C | 0.89 | -0.0288051 | 0.00316571 | 9.11E-20 | 82.79 |
| European | PPARG | rs3105363 | 3 | 12471070 | G | A | 0.24 | 0.0264654 | 0.00248618 | 1.84E-26 | 113.32 |
| European | PPARG | rs310748 | 3 | 12274839 | A | G | 0.28 | 0.0199156 | 0.00244673 | 3.96E-16 | 66.25 |
| European | PPARG | rs310762 | 3 | 12224151 | C | T | 0.43 | 0.0229023 | 0.00205866 | 9.50E-29 | 123.76 |
| European | PPARG | rs35976104 | 3 | 12090463 | A | G | 0.22 | -0.0152003 | 0.00232229 | 5.93E-11 | 42.84 |
| European | PPARG | rs3897889 | 3 | 12754326 | T | C | 0.09 | -0.0190724 | 0.00348188 | 4.31E-08 | 30.00 |
| European | PPARG | rs58691354 | 3 | 12497594 | T | C | 0.20 | 0.0228661 | 0.00280312 | 3.42E-16 | 66.54 |
| European | PPARG | rs66603943 | 3 | 12484681 | C | T | 0.18 | -0.0185979 | 0.00252451 | 1.75E-13 | 54.27 |
| European | PPARG | rs6770851 | 3 | 12797563 | T | C | 0.14 | -0.0223173 | 0.00288442 | 1.02E-14 | 59.86 |
| European | PPARG | rs709154 | 3 | 12456834 | T | A | 0.32 | -0.0298435 | 0.00211705 | 3.98E-45 | 198.72 |
| European | PPARG | rs713178 | 3 | 12615984 | C | T | 0.21 | -0.0142715 | 0.00232576 | 8.45E-10 | 37.65 |
| European | PPARG | rs73130305 | 3 | 12621075 | T | C | 0.19 | -0.0254889 | 0.0024389 | 1.45E-25 | 109.22 |
| European | PPARG | rs73812893 | 3 | 12793837 | T | C | 0.05 | -0.024551 | 0.00442755 | 2.94E-08 | 30.75 |
| European | PPARG | rs7631080 | 3 | 11977950 | A | T | 0.23 | -0.0128081 | 0.00233192 | 3.96E-08 | 30.17 |
| European | PPARG | rs7641345 | 3 | 12296540 | G | A | 0.84 | -0.0183361 | 0.00306141 | 2.11E-09 | 35.87 |
| European | PPARG | rs78512510 | 3 | 12226789 | A | G | 0.03 | -0.0334192 | 0.00537154 | 4.92E-10 | 38.71 |
| European | PPARG | rs79185322 | 3 | 12581266 | A | C | 0.08 | -0.0235996 | 0.00354966 | 2.96E-11 | 44.20 |
| European | PPARG | rs9872031 | 3 | 12496461 | A | G | 0.45 | 0.0238776 | 0.00203975 | 1.19E-31 | 137.03 |
| European | SLC5A2 | rs1060506 | 16 | 31133449 | T | C | 0.33 | 0.0167794 | 0.00222732 | 4.94E-14 | 56.75 |
| European | SLC5A2 | rs1232538 | 16 | 31833812 | T | G | 0.34 | 0.0137088 | 0.00225421 | 1.19E-09 | 36.98 |
| European | SLC5A2 | rs12443808 | 16 | 30996871 | G | C | 0.47 | -0.0155067 | 0.0020814 | 9.32E-14 | 55.50 |
| European | SLC5A2 | rs12932429 | 16 | 31403145 | C | T | 0.91 | -0.0283107 | 0.00468892 | 1.56E-09 | 36.45 |
| European | SLC5A2 | rs17855121 | 16 | 31004169 | C | T | 0.29 | 0.0154563 | 0.00234045 | 4.00E-11 | 43.61 |
| European | SLC5A2 | rs28675289 | 16 | 31463252 | T | C | 0.05 | -0.0384834 | 0.00494068 | 6.75E-15 | 60.67 |
| European | SLC5A2 | rs28692853 | 16 | 31573030 | A | C | 0.44 | -0.0141113 | 0.00201656 | 2.60E-12 | 48.97 |
| European | SLC5A2 | rs45625038 | 16 | 31418975 | T | C | 0.02 | 0.0392602 | 0.00590657 | 2.99E-11 | 44.18 |
| European | SLC5A2 | rs8050500 | 16 | 31404571 | C | T | 0.46 | -0.0261764 | 0.00202389 | 2.90E-38 | 167.28 |
| European | SLC5A2 | rs9923231 | 16 | 31107689 | T | C | 0.37 | -0.0116341 | 0.00207764 | 2.15E-08 | 31.36 |
| European | SLC5A2 | rs9929691 | 16 | 31552196 | T | C | 0.22 | 0.0169729 | 0.00281974 | 1.75E-09 | 36.23 |
| East Asian | ABCC8 | rs1002226 | 11 | 17405617 | C | T | 0.39 | 0.0203 | 0.00435 | 3.20E-06 | 14.01 |
| East Asian | GLP1R | rs35887128 | 6 | 39037858 | A | G | 0.71 | -0.836 | 0.205 | 2.40E-05 | 28530.33 |

**Table S4. Genetic correlation results of instrument for GLP1R single nucleotide polymorphisms among European.**

| RS_number | rs9470964 | rs910166 | rs10305518 | rs6904583 |
| --- | --- | --- | --- | --- |
| rs9470964 | 1 | 0.13 | 0.161 | 0 |
| rs910166 | 0.13 | 1 | 0.127 | 0.005 |
| rs10305518 | 0.161 | 0.127 | 1 | 0 |
| rs6904583 | 0 | 0.005 | 0 | 1 |

**Table S5. Genetic correlation results of instrument for ABCC8 single nucleotide polymorphisms among European.**

| RS_number | rs664382 | rs61880293 | rs2883548 | rs10832777 | rs757110 |
| --- | --- | --- | --- | --- | --- |
| rs664382 | 1 | 0.049 | 0.123 | 0.131 | 0.297 |
| rs61880293 | 0.049 | 1 | 0.038 | 0.052 | 0.134 |
| rs2883548 | 0.123 | 0.038 | 1 | 0.019 | 0.242 |
| rs10832777 | 0.131 | 0.052 | 0.019 | 1 | 0.286 |
| rs757110 | 0.297 | 0.134 | 0.242 | 0.286 | 1 |

**Table S6. Genetic correlation results of instrument for SLC5A2 single nucleotide polymorphisms among European.**

| RS_number | rs12443808 | rs17855121 | rs9923231 | rs1060506 | rs12929023 | rs12932429 | rs8050500 | rs45625038 | rs28675289 | rs9929691 | rs28692853 | rs1232538 |
| --- | --- | --- | --- | --- | --- | --- | --- | --- | --- | --- | --- | --- |
| rs12443808 | 1 | 0.044 | 0.205 | 0.259 | 0.007 | 0.012 | 0.001 | 0.009 | 0.003 | 0 | 0.001 | 0.01 |
| rs17855121 | 0.044 | 1 | 0.174 | 0.132 | 0.085 | 0.008 | 0.004 | 0.011 | 0.003 | 0.003 | 0 | 0 |
| rs9923231 | 0.205 | 0.174 | 1 | 0.202 | 0.023 | 0.006 | 0.003 | 0.003 | 0 | 0 | 0 | 0.01 |
| rs1060506 | 0.259 | 0.132 | 0.202 | 1 | 0.003 | 0.027 | 0 | 0.02 | 0.005 | 0.001 | 0.004 | 0.021 |
| rs12929023 | 0.007 | 0.085 | 0.023 | 0.003 | 1 | 0.005 | 0.054 | 0.002 | 0.001 | 0.003 | 0.011 | 0.001 |
| rs12932429 | 0.012 | 0.008 | 0.006 | 0.027 | 0.005 | 1 | 0.045 | 0.002 | 0.003 | 0.234 | 0.034 | 0.038 |
| rs8050500 | 0.001 | 0.004 | 0.003 | 0 | 0.054 | 0.045 | 1 | 0.03 | 0.06 | 0.063 | 0.129 | 0.003 |
| rs45625038 | 0.009 | 0.011 | 0.003 | 0.02 | 0.002 | 0.002 | 0.03 | 1 | 0.002 | 0.005 | 0.024 | 0.009 |
| rs28675289 | 0.003 | 0.003 | 0 | 0.005 | 0.001 | 0.003 | 0.06 | 0.002 | 1 | 0.009 | 0.052 | 0 |
| rs9929691 | 0 | 0.003 | 0 | 0.001 | 0.003 | 0.234 | 0.063 | 0.005 | 0.009 | 1 | 0.178 | 0.007 |
| rs28692853 | 0.001 | 0 | 0 | 0.004 | 0.011 | 0.034 | 0.129 | 0.024 | 0.052 | 0.178 | 1 | 0.083 |
| rs1232538 | 0.01 | 0 | 0.01 | 0.021 | 0.001 | 0.038 | 0.003 | 0.009 | 0 | 0.007 | 0.083 | 1 |

**Table S7. Genetic correlation results of instrument for PPARG single nucleotide polymorphisms among European.**

| RS_number | rs13320580 | rs7631080 | rs116354045 | rs2600258 | rs17669026 | rs17669062 | rs35976104 | rs307586 | rs115633989 | rs310762 | rs78512510 | rs1822534 | rs17671592 | rs310748 | rs60192719 | rs17036143 | rs7641345 | rs112496224 | rs2920500 | rs568984015 | rs2921186 | rs709154 | rs3105363 | rs66603943 | rs9872031 | rs58691354 | rs13059878 | rs79185322 | rs12492608 | rs713178 | rs2454429 | rs73130305 | rs76071695 | rs3897889 | rs2055740 | rs73812893 | rs6770851 | rs2305398 |
| --- | --- | --- | --- | --- | --- | --- | --- | --- | --- | --- | --- | --- | --- | --- | --- | --- | --- | --- | --- | --- | --- | --- | --- | --- | --- | --- | --- | --- | --- | --- | --- | --- | --- | --- | --- | --- | --- | --- |
| rs13320580 | 1 | 0.034 | 0.018 | 0.037 | 0.001 | 0.039 | 0.097 | 0.028 | 0.005 | 0.008 | 0 | 0.028 | 0.003 | 0.058 | 0 | 0.011 | 0 | 0.008 | 0.096 | 0.008 | 0.024 | 0.036 | 0.017 | 0.021 | 0.018 | 0.006 | 0.001 | 0.007 | 0 | 0 | 0.005 | 0.023 | 0.001 | 0.015 | 0.004 | 0.007 | 0.005 | 0.014 |
| rs7631080 | 0.034 | 1 | 0.057 | 0.066 | 0.001 | 0 | 0.007 | 0.013 | 0.001 | 0.005 | 0.002 | 0.028 | 0.001 | 0.003 | 0.007 | 0.001 | 0.021 | 0.025 | 0.01 | 0.004 | 0.012 | 0.017 | 0.002 | 0.004 | 0.002 | 0 | 0.003 | 0.014 | 0.001 | 0.003 | 0.006 | 0.013 | 0.004 | 0.01 | 0 | 0 | 0.001 | 0 |
| rs116354045 | 0.018 | 0.057 | 1 | 0 | 0.025 | 0.001 | 0.003 | 0 | 0.001 | 0.004 | 0.001 | 0.004 | 0.001 | 0.001 | 0.005 | 0 | 0.001 | 0.15 | 0.002 | 0.002 | 0.003 | 0.01 | 0.002 | 0.003 | 0.001 | 0 | 0.013 | 0.001 | 0 | 0.003 | 0.004 | 0.019 | 0.006 | 0.043 | 0.001 | 0.001 | 0.001 | 0 |
| rs2600258 | 0.037 | 0.066 | 0 | 1 | 0.019 | 0.014 | 0.062 | 0.063 | 0.008 | 0.008 | 0.006 | 0.131 | 0.01 | 0.016 | 0.002 | 0.021 | 0.033 | 0.006 | 0.108 | 0 | 0.012 | 0.033 | 0.003 | 0.038 | 0.042 | 0.002 | 0.002 | 0.004 | 0.001 | 0.002 | 0.008 | 0.008 | 0 | 0.006 | 0.005 | 0.002 | 0.009 | 0.003 |
| rs17669026 | 0.001 | 0.001 | 0.025 | 0.019 | 1 | 0.019 | 0.066 | 0.025 | 0.01 | 0.144 | 0.019 | 0.116 | 0.011 | 0 | 0.007 | 0.029 | 0.032 | 0.15 | 0.03 | 0.005 | 0.022 | 0.052 | 0.038 | 0 | 0.004 | 0.005 | 0.01 | 0 | 0.006 | 0.004 | 0 | 0.014 | 0.003 | 0.019 | 0.003 | 0 | 0.007 | 0.004 |
| rs17669062 | 0.039 | 0 | 0.001 | 0.014 | 0.019 | 1 | 0.271 | 0.01 | 0.004 | 0.051 | 0.003 | 0.109 | 0.005 | 0.025 | 0.021 | 0.003 | 0.007 | 0.004 | 0.058 | 0.006 | 0.011 | 0.11 | 0.014 | 0.094 | 0.031 | 0.013 | 0.003 | 0.086 | 0.002 | 0.023 | 0.018 | 0.027 | 0.001 | 0 | 0.005 | 0.111 | 0.066 | 0.013 |
| rs35976104 | 0.097 | 0.007 | 0.003 | 0.062 | 0.066 | 0.271 | 1 | 0.034 | 0.128 | 0.017 | 0.008 | 0.124 | 0.016 | 0.079 | 0 | 0.03 | 0.036 | 0.012 | 0.241 | 0 | 0.02 | 0.027 | 0.007 | 0.016 | 0.042 | 0.006 | 0.001 | 0.015 | 0 | 0.012 | 0.008 | 0.003 | 0.001 | 0 | 0.005 | 0.023 | 0.016 | 0.01 |
| rs307586 | 0.028 | 0.013 | 0 | 0.063 | 0.025 | 0.01 | 0.034 | 1 | 0.005 | 0.042 | 0.004 | 0.066 | 0.005 | 0.004 | 0.012 | 0.249 | 0.002 | 0.001 | 0.027 | 0.011 | 0.003 | 0.03 | 0.117 | 0.026 | 0.021 | 0.007 | 0.015 | 0.007 | 0 | 0.007 | 0.001 | 0.009 | 0.029 | 0 | 0.042 | 0.005 | 0.008 | 0 |
| rs115633989 | 0.005 | 0.001 | 0.001 | 0.008 | 0.01 | 0.004 | 0.128 | 0.005 | 1 | 0.03 | 0.151 | 0.064 | 0.003 | 0.013 | 0.013 | 0.006 | 0.007 | 0.002 | 0.049 | 0.005 | 0.008 | 0.007 | 0.008 | 0.001 | 0.005 | 0 | 0.027 | 0.004 | 0 | 0 | 0.003 | 0.005 | 0.005 | 0.003 | 0.002 | 0.003 | 0 | 0.001 |
| rs310762 | 0.008 | 0.005 | 0.004 | 0.008 | 0.144 | 0.051 | 0.017 | 0.042 | 0.03 | 1 | 0.022 | 0.275 | 0.041 | 0.141 | 0.217 | 0.033 | 0.119 | 0.028 | 0.012 | 0.037 | 0.102 | 0.12 | 0.041 | 0.001 | 0.014 | 0.027 | 0.019 | 0.019 | 0.01 | 0.012 | 0.028 | 0.034 | 0.003 | 0.005 | 0 | 0.007 | 0.016 | 0.001 |
| rs78512510 | 0 | 0.002 | 0.001 | 0.006 | 0.019 | 0.003 | 0.008 | 0.004 | 0.151 | 0.022 | 1 | 0.046 | 0.002 | 0.009 | 0.01 | 0.005 | 0.005 | 0.001 | 0 | 0.004 | 0.006 | 0.003 | 0.008 | 0.005 | 0.001 | 0 | 0.004 | 0.001 | 0.001 | 0.004 | 0 | 0.01 | 0.003 | 0.001 | 0.002 | 0.002 | 0.002 | 0.002 |
| rs1822534 | 0.028 | 0.028 | 0.004 | 0.131 | 0.116 | 0.109 | 0.124 | 0.066 | 0.064 | 0.275 | 0.046 | 1 | 0.083 | 0.187 | 0.188 | 0.08 | 0.099 | 0.052 | 0.268 | 0.066 | 0.089 | 0.269 | 0.11 | 0.066 | 0.116 | 0.03 | 0.055 | 0.006 | 0.01 | 0.019 | 0.047 | 0.024 | 0.024 | 0.018 | 0.011 | 0.016 | 0.03 | 0.022 |
| rs17671592 | 0.003 | 0.001 | 0.001 | 0.01 | 0.011 | 0.005 | 0.016 | 0.005 | 0.003 | 0.041 | 0.002 | 0.083 | 1 | 0.018 | 0.016 | 0.009 | 0.005 | 0.001 | 0.031 | 0.005 | 0.009 | 0.012 | 0.006 | 0.002 | 0.01 | 0.005 | 0.041 | 0.002 | 0.011 | 0.037 | 0.014 | 0.001 | 0.018 | 0 | 0.003 | 0 | 0.001 | 0.003 |
| rs310748 | 0.058 | 0.003 | 0.001 | 0.016 | 0 | 0.025 | 0.079 | 0.004 | 0.013 | 0.141 | 0.009 | 0.187 | 0.018 | 1 | 0.17 | 0.041 | 0.041 | 0.013 | 0.245 | 0.017 | 0.04 | 0.104 | 0.104 | 0.032 | 0.082 | 0.118 | 0.011 | 0.006 | 0.003 | 0.015 | 0.035 | 0.004 | 0.001 | 0 | 0.003 | 0 | 0.003 | 0.009 |
| rs60192719 | 0 | 0.007 | 0.005 | 0.002 | 0.007 | 0.021 | 0 | 0.012 | 0.013 | 0.217 | 0.01 | 0.188 | 0.016 | 0.17 | 1 | 0.043 | 0.025 | 0.014 | 0.011 | 0.047 | 0.072 | 0.095 | 0.028 | 0.009 | 0.018 | 0.049 | 0.009 | 0.008 | 0 | 0.004 | 0.022 | 0.022 | 0.005 | 0.004 | 0 | 0.004 | 0.007 | 0.003 |
| rs17036143 | 0.011 | 0.001 | 0 | 0.021 | 0.029 | 0.003 | 0.03 | 0.249 | 0.006 | 0.033 | 0.005 | 0.08 | 0.009 | 0.041 | 0.043 | 1 | 0.022 | 0.006 | 0.13 | 0.014 | 0.017 | 0 | 0.052 | 0.013 | 0.053 | 0.014 | 0.02 | 0 | 0.003 | 0.002 | 0.001 | 0 | 0.009 | 0 | 0 | 0.003 | 0.003 | 0.001 |
| rs7641345 | 0 | 0.021 | 0.001 | 0.033 | 0.032 | 0.007 | 0.036 | 0.002 | 0.007 | 0.119 | 0.005 | 0.099 | 0.005 | 0.041 | 0.025 | 0.022 | 1 | 0.007 | 0.018 | 0 | 0.225 | 0.057 | 0.022 | 0.005 | 0.019 | 0.003 | 0 | 0.009 | 0.005 | 0.001 | 0.002 | 0.025 | 0.019 | 0.003 | 0.014 | 0.004 | 0.006 | 0.005 |
| rs112496224 | 0.008 | 0.025 | 0.15 | 0.006 | 0.15 | 0.004 | 0.012 | 0.001 | 0.002 | 0.028 | 0.001 | 0.052 | 0.001 | 0.013 | 0.014 | 0.006 | 0.007 | 1 | 0.034 | 0.005 | 0.009 | 0.053 | 0.011 | 0.004 | 0.014 | 0.002 | 0.062 | 0.004 | 0.009 | 0 | 0.008 | 0.037 | 0.018 | 0.076 | 0.001 | 0.003 | 0.001 | 0 |
| rs2920500 | 0.096 | 0.01 | 0.002 | 0.108 | 0.03 | 0.058 | 0.241 | 0.027 | 0.049 | 0.012 | 0 | 0.268 | 0.031 | 0.245 | 0.011 | 0.13 | 0.018 | 0.034 | 1 | 0.007 | 0.161 | 0.171 | 0.089 | 0.127 | 0.194 | 0.032 | 0.003 | 0.003 | 0.004 | 0.003 | 0.019 | 0.011 | 0 | 0.011 | 0 | 0.008 | 0.017 | 0.016 |
| rs568984015 | 0.008 | 0.004 | 0.002 | 0 | 0.005 | 0.006 | 0 | 0.011 | 0.005 | 0.037 | 0.004 | 0.066 | 0.005 | 0.017 | 0.047 | 0.014 | 0 | 0.005 | 0.007 | 1 | 0.025 | 0.046 | 0.053 | 0.011 | 0.016 | 0.006 | 0.011 | 0 | 0.004 | 0.002 | 0.001 | 0.005 | 0.001 | 0.004 | 0 | 0.001 | 0.002 | 0 |
| rs2921186 | 0.024 | 0.012 | 0.003 | 0.012 | 0.022 | 0.011 | 0.02 | 0.003 | 0.008 | 0.102 | 0.006 | 0.089 | 0.009 | 0.04 | 0.072 | 0.017 | 0.225 | 0.009 | 0.161 | 0.025 | 1 | 0.1 | 0.002 | 0.002 | 0.016 | 0 | 0.021 | 0.011 | 0.002 | 0 | 0.008 | 0.026 | 0.024 | 0.007 | 0.024 | 0.007 | 0.01 | 0 |
| rs709154 | 0.036 | 0.017 | 0.01 | 0.033 | 0.052 | 0.11 | 0.027 | 0.03 | 0.007 | 0.12 | 0.003 | 0.269 | 0.012 | 0.104 | 0.095 | 0 | 0.057 | 0.053 | 0.171 | 0.046 | 0.1 | 1 | 0.118 | 0.159 | 0.086 | 0.02 | 0.012 | 0.032 | 0 | 0 | 0.034 | 0.107 | 0.005 | 0.047 | 0.003 | 0.016 | 0.038 | 0.016 |
| rs3105363 | 0.017 | 0.002 | 0.002 | 0.003 | 0.038 | 0.014 | 0.007 | 0.117 | 0.008 | 0.041 | 0.008 | 0.11 | 0.006 | 0.104 | 0.028 | 0.052 | 0.022 | 0.011 | 0.089 | 0.053 | 0.002 | 0.118 | 1 | 0.055 | 0.115 | 0.037 | 0.016 | 0.01 | 0.006 | 0.041 | 0 | 0.002 | 0.024 | 0 | 0 | 0 | 0.003 | 0.003 |
| rs66603943 | 0.021 | 0.004 | 0.003 | 0.038 | 0 | 0.094 | 0.016 | 0.026 | 0.001 | 0.001 | 0.005 | 0.066 | 0.002 | 0.032 | 0.009 | 0.013 | 0.005 | 0.004 | 0.127 | 0.011 | 0.002 | 0.159 | 0.055 | 1 | 0.122 | 0.015 | 0.102 | 0.079 | 0.001 | 0.008 | 0.021 | 0.001 | 0.028 | 0.01 | 0.006 | 0.069 | 0.015 | 0.032 |
| rs9872031 | 0.018 | 0.002 | 0.001 | 0.042 | 0.004 | 0.031 | 0.042 | 0.021 | 0.005 | 0.014 | 0.001 | 0.116 | 0.01 | 0.082 | 0.018 | 0.053 | 0.019 | 0.014 | 0.194 | 0.016 | 0.016 | 0.086 | 0.115 | 0.122 | 1 | 0.264 | 0.242 | 0.015 | 0.016 | 0.06 | 0.066 | 0.048 | 0.019 | 0.034 | 0 | 0.014 | 0.042 | 0.019 |
| rs58691354 | 0.006 | 0 | 0 | 0.002 | 0.005 | 0.013 | 0.006 | 0.007 | 0 | 0.027 | 0 | 0.03 | 0.005 | 0.118 | 0.049 | 0.014 | 0.003 | 0.002 | 0.032 | 0.006 | 0 | 0.02 | 0.037 | 0.015 | 0.264 | 1 | 0.064 | 0.015 | 0.001 | 0.04 | 0.169 | 0.036 | 0.001 | 0.014 | 0.012 | 0.005 | 0.014 | 0.027 |
| rs13059878 | 0.001 | 0.003 | 0.013 | 0.002 | 0.01 | 0.003 | 0.001 | 0.015 | 0.027 | 0.019 | 0.004 | 0.055 | 0.041 | 0.011 | 0.009 | 0.02 | 0 | 0.062 | 0.003 | 0.011 | 0.021 | 0.012 | 0.016 | 0.102 | 0.242 | 0.064 | 1 | 0.018 | 0.018 | 0.036 | 0.083 | 0.086 | 0.125 | 0.136 | 0.01 | 0.012 | 0.017 | 0.003 |
| rs79185322 | 0.007 | 0.014 | 0.001 | 0.004 | 0 | 0.086 | 0.015 | 0.007 | 0.004 | 0.019 | 0.001 | 0.006 | 0.002 | 0.006 | 0.008 | 0 | 0.009 | 0.004 | 0.003 | 0 | 0.011 | 0.032 | 0.01 | 0.079 | 0.015 | 0.015 | 0.018 | 1 | 0.018 | 0.243 | 0.053 | 0.291 | 0.07 | 0.002 | 0.005 | 0.305 | 0.139 | 0.001 |
| rs12492608 | 0 | 0.001 | 0 | 0.001 | 0.006 | 0.002 | 0 | 0 | 0 | 0.01 | 0.001 | 0.01 | 0.011 | 0.003 | 0 | 0.003 | 0.005 | 0.009 | 0.004 | 0.004 | 0.002 | 0 | 0.006 | 0.001 | 0.016 | 0.001 | 0.018 | 0.018 | 1 | 0.064 | 0.021 | 0.017 | 0.005 | 0.005 | 0 | 0.016 | 0.017 | 0 |
| rs713178 | 0 | 0.003 | 0.003 | 0.002 | 0.004 | 0.023 | 0.012 | 0.007 | 0 | 0.012 | 0.004 | 0.019 | 0.037 | 0.015 | 0.004 | 0.002 | 0.001 | 0 | 0.003 | 0.002 | 0 | 0 | 0.041 | 0.008 | 0.06 | 0.04 | 0.036 | 0.243 | 0.064 | 1 | 0.202 | 0.017 | 0.175 | 0.013 | 0.016 | 0.071 | 0.039 | 0.002 |
| rs2454429 | 0.005 | 0.006 | 0.004 | 0.008 | 0 | 0.018 | 0.008 | 0.001 | 0.003 | 0.028 | 0 | 0.047 | 0.014 | 0.035 | 0.022 | 0.001 | 0.002 | 0.008 | 0.019 | 0.001 | 0.008 | 0.034 | 0 | 0.021 | 0.066 | 0.169 | 0.083 | 0.053 | 0.021 | 0.202 | 1 | 0.19 | 0.009 | 0.075 | 0.06 | 0.015 | 0.025 | 0.113 |
| rs73130305 | 0.023 | 0.013 | 0.019 | 0.008 | 0.014 | 0.027 | 0.003 | 0.009 | 0.005 | 0.034 | 0.01 | 0.024 | 0.001 | 0.004 | 0.022 | 0 | 0.025 | 0.037 | 0.011 | 0.005 | 0.026 | 0.107 | 0.002 | 0.001 | 0.048 | 0.036 | 0.086 | 0.291 | 0.017 | 0.017 | 0.19 | 1 | 0.13 | 0.284 | 0.013 | 0.123 | 0.131 | 0.022 |
| rs76071695 | 0.001 | 0.004 | 0.006 | 0 | 0.003 | 0.001 | 0.001 | 0.029 | 0.005 | 0.003 | 0.003 | 0.024 | 0.018 | 0.001 | 0.005 | 0.009 | 0.019 | 0.018 | 0 | 0.001 | 0.024 | 0.005 | 0.024 | 0.028 | 0.019 | 0.001 | 0.125 | 0.07 | 0.005 | 0.175 | 0.009 | 0.13 | 1 | 0.072 | 0.008 | 0.023 | 0.03 | 0.004 |
| rs3897889 | 0.015 | 0.01 | 0.043 | 0.006 | 0.019 | 0 | 0 | 0 | 0.003 | 0.005 | 0.001 | 0.018 | 0 | 0 | 0.004 | 0 | 0.003 | 0.076 | 0.011 | 0.004 | 0.007 | 0.047 | 0 | 0.01 | 0.034 | 0.014 | 0.136 | 0.002 | 0.005 | 0.013 | 0.075 | 0.284 | 0.072 | 1 | 0.007 | 0.007 | 0.027 | 0.016 |
| rs2055740 | 0.004 | 0 | 0.001 | 0.005 | 0.003 | 0.005 | 0.005 | 0.042 | 0.002 | 0 | 0.002 | 0.011 | 0.003 | 0.003 | 0 | 0 | 0.014 | 0.001 | 0 | 0 | 0.024 | 0.003 | 0 | 0.006 | 0 | 0.012 | 0.01 | 0.005 | 0 | 0.016 | 0.06 | 0.013 | 0.008 | 0.007 | 1 | 0.003 | 0.007 | 0.067 |
| rs73812893 | 0.007 | 0 | 0.001 | 0.002 | 0 | 0.111 | 0.023 | 0.005 | 0.003 | 0.007 | 0.002 | 0.016 | 0 | 0 | 0.004 | 0.003 | 0.004 | 0.003 | 0.008 | 0.001 | 0.007 | 0.016 | 0 | 0.069 | 0.014 | 0.005 | 0.012 | 0.305 | 0.016 | 0.071 | 0.015 | 0.123 | 0.023 | 0.007 | 0.003 | 1 | 0.303 | 0.014 |
| rs6770851 | 0.005 | 0.001 | 0.001 | 0.009 | 0.007 | 0.066 | 0.016 | 0.008 | 0 | 0.016 | 0.002 | 0.03 | 0.001 | 0.003 | 0.007 | 0.003 | 0.006 | 0.001 | 0.017 | 0.002 | 0.01 | 0.038 | 0.003 | 0.015 | 0.042 | 0.014 | 0.017 | 0.139 | 0.017 | 0.039 | 0.025 | 0.131 | 0.03 | 0.027 | 0.007 | 0.303 | 1 | 0.034 |
| rs2305398 | 0.014 | 0 | 0 | 0.003 | 0.004 | 0.013 | 0.01 | 0 | 0.001 | 0.001 | 0.002 | 0.022 | 0.003 | 0.009 | 0.003 | 0.001 | 0.005 | 0 | 0.016 | 0 | 0 | 0.016 | 0.003 | 0.032 | 0.019 | 0.027 | 0.003 | 0.001 | 0 | 0.002 | 0.113 | 0.022 | 0.004 | 0.016 | 0.067 | 0.014 | 0.034 | 1 |

**Table S8. Mendelian randomization analysis of genetic instruments for positive control (Estimates are effects equivalent to 1 standard deviation increment in HbA1c).**

| **Ancestry** | **Target** | **Outcome** | **SNPs** | **Method** | **OR (95% CI)** | **P value** |
| --- | --- | --- | --- | --- | --- | --- |
| European | ABCC8 | Type 2 diabetes | 5 | IVW | 15.70 (10.73-22.95) | 8.62E-46 |
| European | GLP1R | Type 2 diabetes | 4 | IVW | 3.755 (2.401-5.872) | 6.73E-09 |
| European | PPARG | Type 2 diabetes | 32 | IVW | 1.865 (1.569-2.217) | 1.57E-12 |
| European | SLC5A2 | Type 2 diabetes | 11 | IVW | 1.491 (1.277-1.741) | 4.22E-07 |
| East Asian | ABCC8 | Type 2 diabetes | 1 | Wald ratio | 44.39 (23.47-83.96) | 1.89E-31 |
| East Asian | GLP1R | Type 2 diabetes | 1 | Wald ratio | 1.017 (0.913-1.133) | 0.763 |
| European | ABCC8 | Body mass index | 3 | IVW | 1.748 (1.502-2.037) | 7.01E-13 |
| European | GLP1R | Body mass index | 2 | IVW | 0.810 (0.665-0.986) | 0.036 |
| European | PPARG | Alanine aminotransferase levels | 33 | IVW | 0.668 (0.623-0.718) | 1.04E-05 |
| European | PPARG | Aspartate aminotransferase levels | 33 | IVW | 0.710 (0.657-0.766) | 6.15E-04 |
| East Asian | ABCC8 | Body mass index | 1 | Wald ratio | 2.197 (1.598-3.020) | 1.27E-06 |
| East Asian | GLP1R | Body mass index | 1 | Wald ratio | 0.718 (0.510-1.010) | 0.057 |

Notes: OR = odds ratio; SNP = single nucleotide polymorphism; IVW = inverse variance-weighted; CI = confidence interval.

| Target | Method | SNPs | IGGC | | GBMI | | FinnGen (R11) | |
| --- | --- | --- | --- | --- | --- | --- | --- | --- |
|  |  |  | OR (95% CI) | P value | OR (95% CI) | P value | OR (95% CI) | P value |
| ABCC8 | MR-Egger | 5 | 0.378 (0.025 - 5.837) | 0.486 | 0.903 (0.064 - 12.71) | 0.940 | 2.255 (0.087 - 58.59) | 0.625 |
|  | IVW | 5 | 0.152 (0.075 - 0.308) | 1.75E-07 | 0.332 (0.154 - 0.716) | 0.0049 | 0.189 (0.071 - 0.500) | 7.96E-04 |
|  | Weighted median | 5 | 0.211 (0.078 - 0.573) | 0.002 | 0.463 (0.181 - 1.180) | 0.107 | 0.244 (0.077 - 0.770) | 0.016 |
|  | Weighted mode | 5 | 0.227 (0.077 - 0.669) | 0.055 | 0.491 (0.161 - 1.495) | 0.279 | 0.265 (0.072 - 0.977) | 0.117 |
|  | Simple mode | 5 | 0.207 (0.055 - 0.786) | 0.082 | 0.461 (0.122 - 1.732) | 0.315 | 0.217 (0.047 - 1.116) | 0.141 |
| GLP1R | MR-Egger | 4 | 11.50 (0.028 - 4768.053) | 0.510 | 2.843 (0.001- 11795.727) | 0.847 | 0.919 (0.001 - 654.896) | 0.984 |
|  | IVW | 4 | 0.496 (0.173 - 1.418) | 0.191 | 0.382 (0.097 - 1.501) | 0.168 | 1.989 (0.488 - 8.113) | 0.337 |
|  | Weighted median | 4 | 0.502 (0.153 - 1.647) | 0.256 | 0.355 (0.075 - 1.686) | 0.192 | 1.418 (0.268 - 7.500) | 0.681 |
|  | Weighted mode | 4 | 0.493 (0.094 - 2.595) | 0.465 | 0.297 (0.039 - 2.263) | 0.362 | 1.320 (0.171 - 10.201) | 0.815 |
|  | Simple mode | 4 | 0.545 (0.102 - 2.913) | 0.529 | 0.312 (0.041 - 2.362) | 0.376 | 1.358 (0.163 - 11.308) | 0.804 |
| PPARG | MR-Egger | 33 | 0.673 (0.269 - 1.683) | 0.403 | 0.780 (0.196 - 3.097) | 0.726 | 1.157 (0.473 - 2.830) | 0.752 |
|  | IVW | 33 | 0.875 (0.655 - 1.168) | 0.365 | 0.512 (0.075 - 1.009) | 0.056 | 0.908 (0.679 - 1.214) | 0.515 |
|  | Weighted median | 33 | 0.869 (0.589 - 1.281) | 0.477 | 0.752 (0.482 - 1.173) | 0.209 | 0.997 (0.662 - 1.501) | 0.987 |
|  | Weighted mode | 33 | 0.804 (0.485 - 1.333) | 0.404 | 0.815 (0.337 - 1.970) | 0.653 | 1.101 (0.554 - 2.186) | 0.786 |
|  | Simple mode | 33 | 0.730 (0.335 - 1.588) | 0.432 | 0.440 (0.179 - 1.080) | 0.083 | 1.026 (0.610 - 1.726) | 0.922 |
| SLC5A2 | MR-Egger | 11 | 2.665 (0.564 - 12.604) | 0.244 | 3.793 (0.674 - 21.337) | 0.191 | 1.933 (0.346 - 10.802) | 0.470 |
|  | IVW | 11 | 1.131 (0.680 - 1.880) | 0.636 | 1.027 (0.583 - 1.808) | 0.926 | 1.166 (0.669 - 2.034) | 0.588 |
|  | Weighted median | 11 | 1.030 (0.528 - 2.010) | 0.931 | 0.917 (0.427 - 1.972) | 0.825 | 1.001 (0.478 - 2.099) | 0.997 |
|  | Weighted mode | 11 | 1.174 (0.482 - 2.855) | 0.731 | 0.721 (0.215 - 2.414) | 0.615 | 0.940 (0.370 - 2.385) | 0.898 |
|  | Simple mode | 11 | 1.960 (0.643 - 5.969) | 0.261 | 0.837 (0.327 - 2.141) | 0.724 | 1.206 (0.356 - 4.086) | 0.769 |

**Table S9. Association between genetically proxied anti-diabetic drug targets and primary open-angle glaucoma in Europeans (Estimates are effects equivalent to 1 standard deviation increment in HbA1c).**

Notes: OR = odds ratio; SNP = single nucleotide polymorphism; IVW = inverse variance-weighted; CI = confidence interval.

**Table S10. Heterogeneity, pleiotropy and MR-PRESSO tests.**

| **Ancestry** | **Target** | **Outcome** | **Q** | **Q_pval** | **egger_intercept** | **egger_intercept_pval** | **correct_causal_direction** | **steiger_pval** | **MR-PRESSO_Global_Test_pval** |
| --- | --- | --- | --- | --- | --- | --- | --- | --- | --- |
| European | ABCC8 | Type 2 diabetes | 10.273 | 0.016 | -0.002 | 0.904 | TRUE | 2.78E-05 | 0.371 |
| European | GLP1R | Type 2 diabetes | 6.716 | 0.814 | 0.061 | 0.123 | TRUE | 2.86E-12 | 0.303 |
| European | PPARG | Type 2 diabetes | 163.330 | 6.55E-20 | -0.002 | 0.758 | TRUE | 9.44E-265 | 0.002 |
| European | SLC5A2 | Type 2 diabetes | 10.191 | 0.335 | 0.010 | 0.043 | TRUE | 1.03E-87 | 0.411 |
| East Asian | ABCC8 | Type 2 diabetes | NA | NA | NA | NA | FALSE | 0.898 | NA |
| East Asian | GLP1R | Type 2 diabetes | NA | NA | NA | NA | TRUE | 1.51E-04 | NA |
| European | ABCC8 | Body mass index | 3.229 | 0.199 | -0.0064 | 0.323 | TRUE | 3.04E-08 | NA |
| European | GLP1R | Body mass index | 5.100 | 0.475 | NA | NA | TRUE | 2.18E-08 | NA |
| European | PPARG | Alanine aminotransferase levels | 8.361 | 2.82E-06 | -0.00036 | 0.879 | TRUE | 4.83E-92 | <0.001 |
| European | PPARG | Aspartate aminotransferase levels | 9.143 | 2.15E-07 | 0.0012 | 0.639 | TRUE | 1.25E-104 | <0.001 |
| East Asian | ABCC8 | Body mass index | NA | NA | NA | NA | TRUE | 0.030 | NA |
| East Asian | GLP1R | Body mass index | NA | NA | NA | NA | TRUE | 0.0015 | NA |
| European | ABCC8 | POAG (IGGC) | 2.278 | 0.684 | 0.016 | 0.498 | TRUE | 6.55E-09 | 0.729 |
| European | ABCC8 | POAG (GBMI) | 2.583 | 0.629 | 0.017 | 0.438 | TRUE | 1.06E-34 | 0.661 |
| European | ABCC8 | POAG (FinnGen) | 3.411 | 0.491 | 0.041 | 0.118 | TRUE | 1.33E-32 | 0.551 |
| East Asian | ABCC8 | POAG (IGGC) | NA | NA | NA | NA | TRUE | 0.116 | NA |
| East Asian | ABCC8 | POAG (GBMI) | NA | NA | NA | NA | TRUE | 0.0027 | NA |
| European | ABCC8 | Intra-ocular pressure, Goldmann-correlated (right eye) | 5.457 | 0.243 | 0.017 | 0.184 | TRUE | 0.601 | 0.376 |
| European | ABCC8 | Intra-ocular pressure, Goldmann-correlated (left eye) | 4.936 | 0.293 | 0.019 | 0.158 | TRUE | 0.011 | 0.428 |

**Table S11. Power calculations in Mendelian randomization study of ABCC8 and risk of primary open-angle glaucoma.**

| **Ancestry** | **Outcome** | **Sample size** | **Proportion of cases** | **Selected scenarios*** | | | | |
| --- | --- | --- | --- | --- | --- | --- | --- | --- |
|  |  |  |  | **OR = 0.80** | **OR = 0.65** | **OR = 0.50** | **OR = 0.35** | **OR = 0.20** |
| Europeans | POAG (IGGC) | 216257 | 0.077 | 0.13 | 0.29 | 0.54 | 0.78 | 0.93 |
| Europeans | POAG (GBMI) | 1172905 | 0.014 | 0.13 | 0.29 | 0.52 | 0.75 | 0.90 |
| Europeans | POAG (FinnGen) | 439815 | 0.022 | 0.09 | 0.19 | 0.34 | 0.63 | 0.81 |
| East Asians | POAG (IGGC) | 46523 | 0.149 | 0.13 | 0.25 | 0.47 | 0.64 | 0.84 |
| East Asians | POAG (GBMI) | 269960 | 0.036 | 0.08 | 0.19 | 0.35 | 0.54 | 0.72 |

Note: *The proportion of variance explained was based on ethnic-specific instrumens and Type 1 error was 5%. OR = odds ratio; GBMI = Global Biobank Meta-analysis Initiative; IGGC = international Glaucoma Genetics Consortium; POAG = primary open-angle glaucoma.

**Table S12. Effect estimates of the associations between ABCC8 perturbation and glaucoma endophenotypes.**

| **Target** | **Outcome** | **SNPs** | **Method** | **OR (95% CI)** | **P value** |
| --- | --- | --- | --- | --- | --- |
| ABCC8 | Intra-ocular pressure, Goldmann-correlated (right eye) | 5 | IVW | 0.581 (0.633-0.785) | 0.004 |
| ABCC8 | Intra-ocular pressure, Goldmann-correlated (left eye) | 5 | IVW | 0.382 (0.712-0.880) | 1.66E-06 |
| ABCC8 | Intra-ocular pressure, Goldmann-correlated (combine) * | 5 | IVW | 0.474 (0.314-0.714) | 3.71E-04 |
| ABCC8 | Optic cup area | 5 | IVW | 1.161 (0.998-1.349) | 0.524 |
| ABCC8 | Optic disc area | 5 | IVW | 1.269 (0.968-1.664) | 0.842 |
| ABCC8 | Vertical cup-to-disc ratio | 5 | IVW | 1.060 (0.977-1.151) | 0.159 |
| ABCC8 | Retinal nerve fiber layer thickness | 5 | IVW | 0.002 (0.000152-0.034) | 1.04E-05 |

Notes: *The combine association was calculated using an inverse variance random-effects meta-analysis of right eye and left eye estimates of effect. OR = odds ratio; SNP = single nucleotide polymorphism; IVW = inverse variance-weighted; CI = confidence interval.

**Table S13. Association between genetically proxied anti-diabetic drug targets and primary open-angle glaucoma in East Asians.**

| Target | Method | SNPs | IGGC | | GBMI | |
| --- | --- | --- | --- | --- | --- | --- |
|  |  |  | OR (95% CI) | P value | OR (95% CI) | P value |
| ABCC8 | Wald ratio | 1 | 0.101 (0.005 - 2.189) | 0.144 | 0.056 (0.005 - 0.605) | 0.0176 |
| GLP1R | Wald ratio | 1 | 0.956 (0.648 - 1.411) | 0.822 | 0.978 (0.646 - 1.479) | 0.916 |

Note: IGGC = international Glaucoma Genetics Consortium; GBMI = Global Biobank Meta-analysis Initiative; OR = odds ratio; CI = confidence interval;

**Table S14. Effect estimates of the associations between genetically predicted intra-ocular pressure and risk of primary open-angle glaucoma.**

| **Exposure** | **Outcome** | **SNPs** | **Method** | **OR (95% CI)** | **P value** |
| --- | --- | --- | --- | --- | --- |
| Intra-ocular pressure, Goldmann-correlated (right eye) | POAG (GBMI) | 56 | IVW | 3.781 (2.978-4.800) | 8.83E-28 |
| Intra-ocular pressure, Goldmann-correlated (right eye) | POAG (IGGC) | 50 | IVW | 4.340 (3.156-5.969) | 1.73E-19 |
| Intra-ocular pressure, Goldmann-correlated (right eye) | POAG (FinnGen) | 54 | IVW | 5.348 (4.047-7.067) | 4.36E-32 |
| Intra-ocular pressure, Goldmann-correlated (left eye) | POAG (GBMI) | 59 | IVW | 3.469 (2.721-4.422) | 1.01E-23 |
| Intra-ocular pressure, Goldmann-correlated (left eye) | POAG (IGGC) | 50 | IVW | 3.770 (2.703-5.257) | 5.27E-15 |
| Intra-ocular pressure, Goldmann-correlated (left eye) | POAG (FinnGen) | 56 | IVW | 4.661 (3.492-6.224) | 1.64E-25 |
| Intra-ocular pressure, Goldmann-correlated (combine) | POAG (GBMI+IGGC+FinnGen) |  | random-effects meta-analysis | 4.126 (3.685 - 4.620 | 2.06E-133 |

Notes: *The combine association was calculated using an inverse variance random-effects meta-analysis of right eye and left eye estimates of effect. OR = odds ratio; SNP = single nucleotide polymorphism; IVW = inverse variance-weighted; CI = confidence interval; IGGC = international Glaucoma Genetics Consortium; GBMI = Global Biobank Meta-analysis Initiative; POAG = primary open-angle glaucoma.

**Table S15. Effect estimates of the associations between ABCC8 perturbation and intraocular pressure.**

| Target | Method | SNPs | Intra-ocular pressure, Goldmann-correlated (right eye) | | Intra-ocular pressure, Goldmann-correlated (left eye) | |
| --- | --- | --- | --- | --- | --- | --- |
|  |  |  | OR (95% CI) | P value | OR (95% CI) | P value |
| ABCC8 | MR-Egger | 5 | 0.997 (0.335-3.004) | 0.996 | 1.728 (0.587-5.089) | 0.320 |
|  | IVW | 5 | 0.382 (0.257-0.566) | 1.66E-06 | 0.581 (0.401-0.841) | 0.004 |
|  | Weighted median | 5 | 0.433 (0.283-0.664) | 1.25E-04 | 0.549 (0.369-0.816) | 0.003 |
|  | Weighted mode | 5 | 0.465 (0.296-0.732) | 0.029 | 0.565 (0.352-1.105) | 0.076 |
|  | Simple mode | 5 | 0.499 (0.276-1.107) | 0.083 | 0.420 ( 0.226-1.278) | 0.052 |

Note: OR = odds ratio; SNP = single nucleotide polymorphism; IVW = inverse variance-weighted; CI = confidence interval.

**Figure S1**. **Scatter plots of the Mendelian randomization analyses for genetically proxied ABCC8 inhibition with the risk of primary open-angle glaucoma. (A) POAG outcome from IGGC GWAS, (B) POAG outcome from GBMI GWAS, (C) POAG outcome from FinnGen GWAS.**


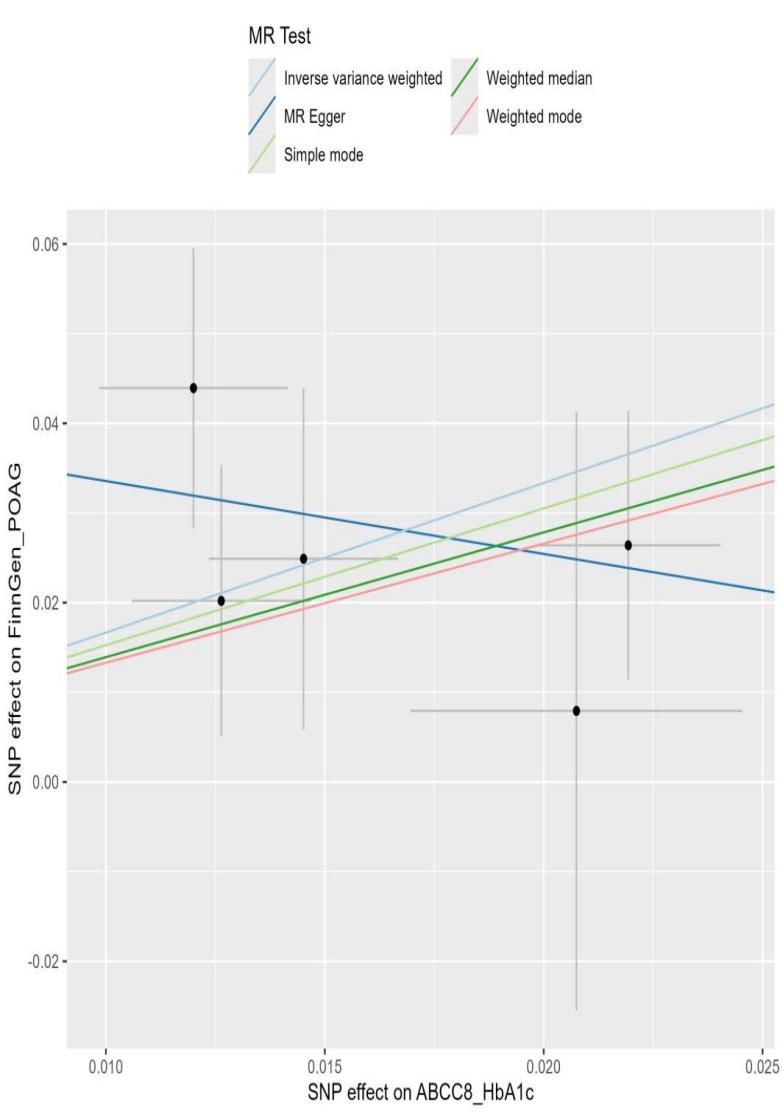

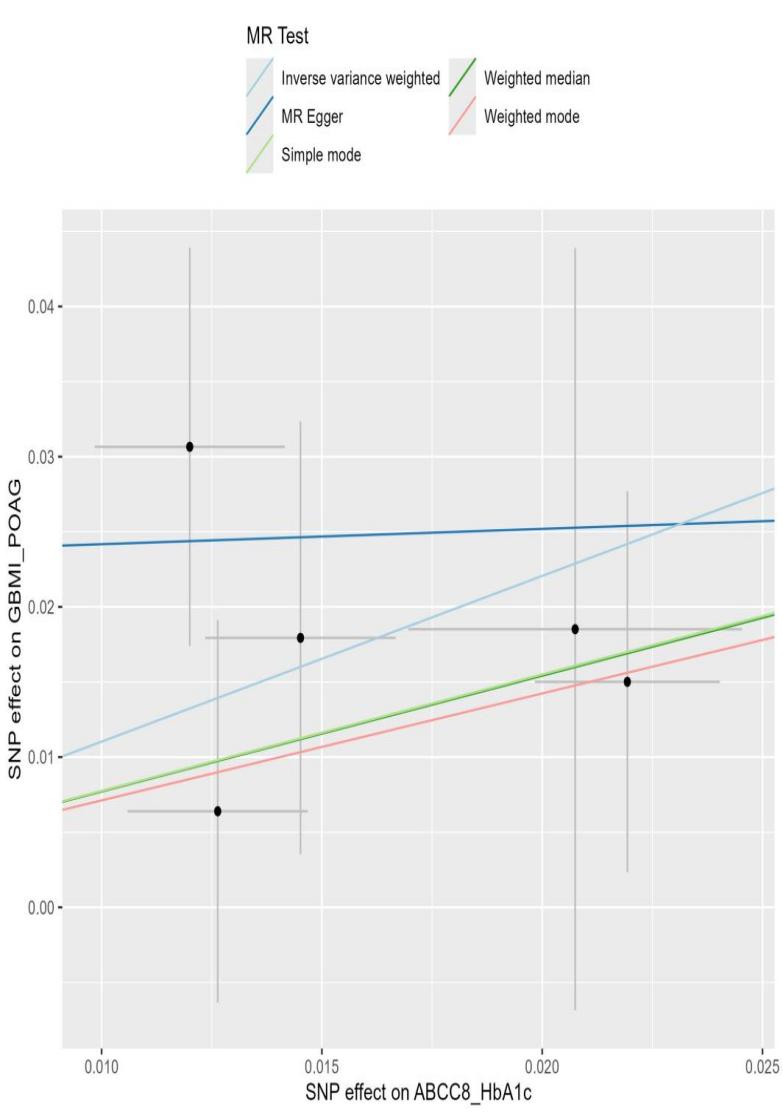

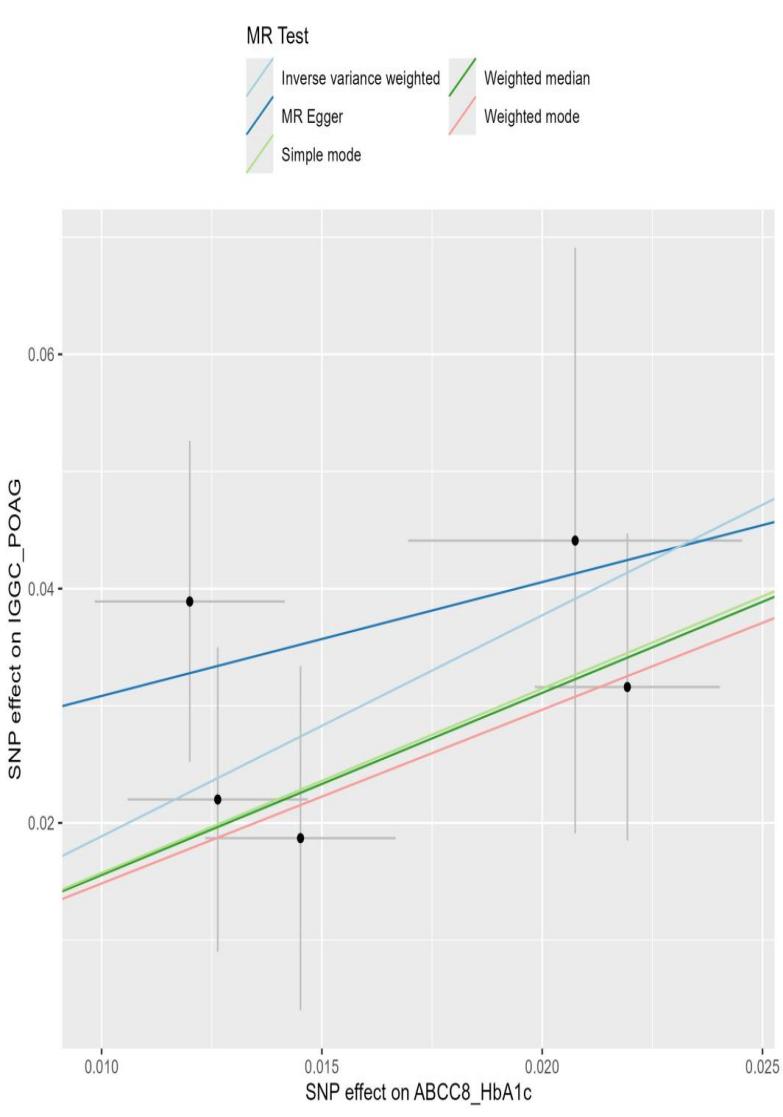


A

B

C

**Figure S2**. **Forest plot for leave-one-out analysis of ABCC8 on primary open-angle glaucoma, with each point denoting the causal effect by IVW after removing the specific SNP. (A) POAG outcome from IGGC GWAS, (B) POAG outcome from GBMI GWAS, (C) POAG outcome from FinnGen GWAS.**


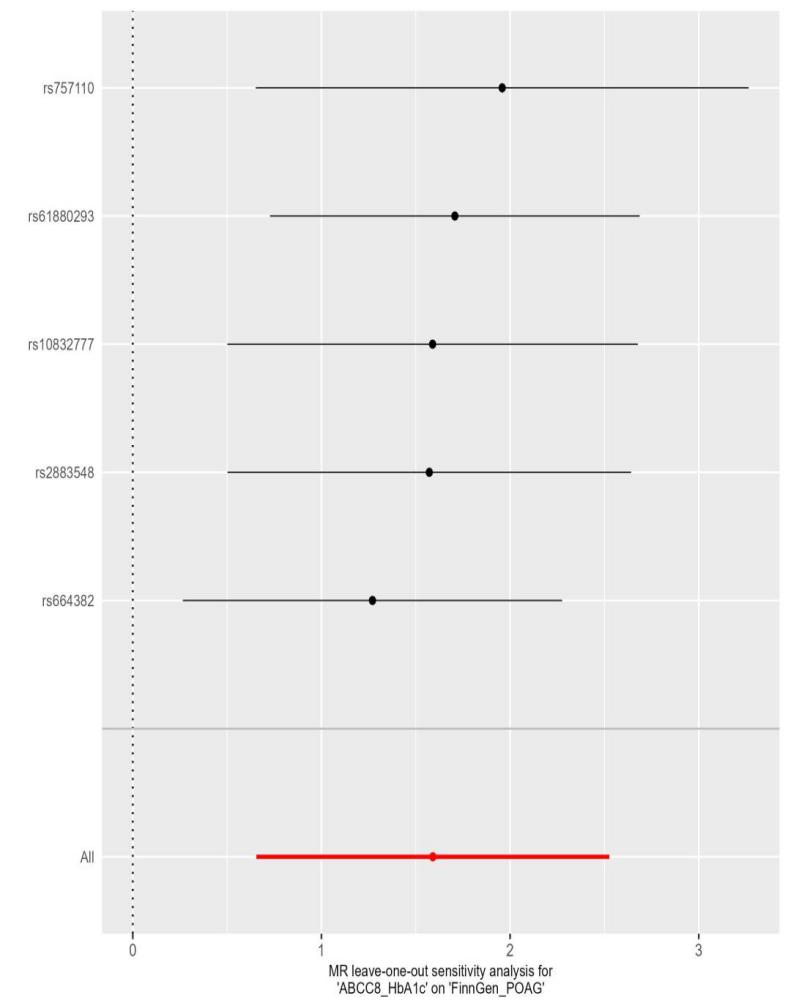

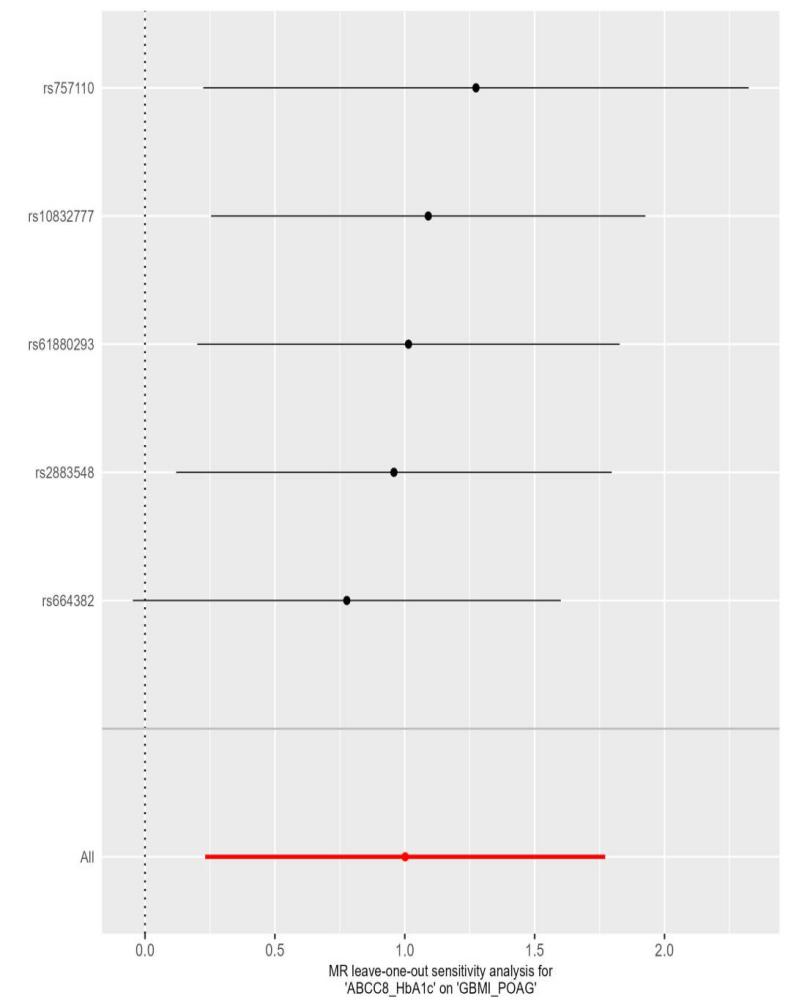

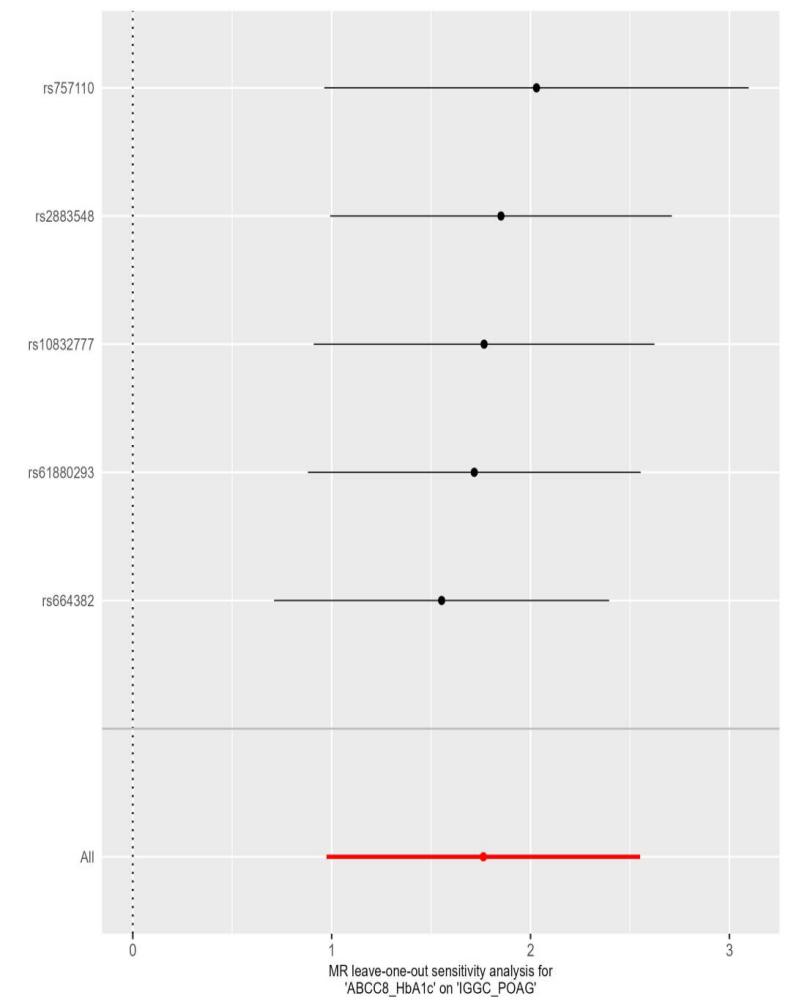


A

B

C
